# Molecular Mechanisms and Experimental Strategies for Understanding Plant Drought Response

**DOI:** 10.3390/plants15010149

**Published:** 2026-01-04

**Authors:** Adrianna Michalak, Karolina Małas, Kinga Dąbrowska, Kinga Półrolniczak, Lidia Bronowska, Anna Misiewicz, Angelika Maj, Maja Stabrowska, Iga Wnuk, Katarzyna Kabała

**Affiliations:** Department of Plant Molecular Physiology, Faculty of Biological Science, University of Wroclaw, Kanonia 6/8, 50-328 Wrocław, Poland; adrianna.michalak2@uwr.edu.pl (A.M.); karolina.malas2@uwr.edu.pl (K.M.);

**Keywords:** drought tolerance, abiotic stress, plant growth, ROS, water relations

## Abstract

Drought severely limits plant growth, threatening global food security and biodiversity. This review provides a comprehensive overview of the recent advances in plant responses to drought, ranging from initial sensing to physiological adaptation, as well as guidelines for experimental design. We focus on key regulatory components, specifically the ABA signaling core (PYR/PYL/RCARs, PP2C phosphatases, and SnRK2 kinases) and ROS signaling. We provide a detailed description of transcriptional networks, highlighting the pivotal roles of DREB, NAC, and MYB transcription factors in coordinating gene expression. Furthermore, we explore downstream tolerance strategies, including osmoprotectant (e.g., proline) accumulation, cell wall remodeling involving expansins and pectin methylesterases, as well as stomatal regulation. We also discuss how combining genetics with multi-omics and high-throughput phenotyping bridges the gap between molecular mechanisms and whole-plant physiological performance. Ultimately, these insights provide a foundation for refining research approaches and accelerating the development of drought-resilient crops to sustain agricultural productivity and ecosystem stability in increasingly arid environments.

## 1. Introduction

Drought represents one of the most damaging environmental constraints for plant growth and global food security [1,2]. The severity of this threat is rapidly escalating due to climate change, which is altering rainfall patterns and increasing the frequency of extreme weather events [3]. The latest climate data underscores the urgency of this issue; global assessments indicate that the land area affected by extreme drought has nearly tripled since the 1980s, with 48% of the Earth’s land surface experiencing at least one month of extreme drought in 2023 [4]. Furthermore, 2024 was reported as the hottest year on record, with the global average temperatures exceeding 1.5 °C above pre-industrial levels for the first time [5]. Recent projections suggest that 2025 will rank among the top three warmest years, with the average temperature from January to August 2025 approximately 1.42 °C higher than pre-industrial levels, according to the World Meteorological Organization [6]. The observed temperature increase in recent years has led to severe agricultural and humanitarian crises; the Drought Hotspots 2023–2025 report notes, for example, that drought caused a 70% decline in corn production in Zimbabwe and a 44% decrease in Malawi. These crop failures left approximately 40% of the population in Malawi and 30% in Zambia facing acute hunger or malnutrition by early 2024 [7]. Understanding the mechanisms of plant resilience is therefore critical for sustaining productivity in these increasingly arid environments [8,9,10,11].

To survive water deficits, plants have evolved intricate strategies, broadly categorized into drought escape, avoidance, and tolerance [1,12]. Drought escape enables plants to complete their life cycle before severe water stress occurs [13], while drought avoidance minimizes water loss by closing stomata or maximizes its uptake via root deeping [11,12]. Drought tolerance, however, involves complex molecular reprogramming to maintain cellular function at low water potential through osmotic adjustment and antioxidant defense.

Despite the large amount of data generated recently, a significant gap in research remains between molecular biology and whole-plant physiology [14]. Furthermore, the lack of consistent physiological standards across these disciplines as well as the multi-scale approach hinders the translation of fundamental genetic discoveries into field-applicable crop traits [15]. This review aims to bridge these disparities by synthesizing fundamental knowledge with the recent advances in molecular mechanisms underlying plant responses to drought. By integrating genetics, physiology, and high-throughput omics, we provide a comprehensive guide for refining experimental strategies and developing drought-resistant crops.

## 2. Sensing and Signaling of Water Stress

In response to environmental stress cues such as drought, plants activate a variety of signaling pathways including hydraulic waves, Ca^2+^, reactive oxygen species (ROS), small peptides, and phytohormones, to coordinate complex physiological and molecular processes [16,17]. Together, these pathways play a fundamental role in orchestrating the plant’s intricate response to water stress [17,18,19].

### 2.1. Local Sensing and Signaling Pathways

Although drought sensing and signaling are complex processes that are not yet fully elucidated, current knowledge allows us to reconstruct a probable sequence of events in the plant’s response to water stress. Under drought conditions, plants perceive alterations in their cellular water status, initiating a cascade of physiological and molecular changes [20]. In plant cells, the water potential (Ψ_w_) is governed by a fundamental thermodynamic relationship: Ψ_w_ = Ψ_s_ + Ψ_p_. Ψ_s_ (osmotic potential) reflects the concentration of solutes in cells (always negative), acting to lower Ψ_w_ and attract water, while Ψ_p_ (turgor pressure) represents the physical hydrostatic pressure pushing against the cell wall (typically positive). The water relations between any two systems, such as neighboring plant cells, are determined by their Ψ_w_ values, as water flows passively from areas of higher (less negative) potential to areas of lower (more negative) potential [21]. Soil and atmosphere are also characterized by Ψ_w_ values. The soil Ψ_w_ depends primarily on the matric potential (Ψ_m_), the force binding water to soil particles, and the solute concentration (Ψ_s_), while the atmospheric Ψ_w_ is determined by water vapor pressure [22]. Under non-stress conditions, the Ψ_w_ values within the Soil–Plant-Atmosphere Continuum (SPAC) form a gradient (Ψ_soil_ > Ψ_plant_ > Ψ_atmosphere_), which drives passive water uptake [23]. However, drought disrupts this equilibrium. Loss of water via evaporation strengthens the binding of the remaining water to soil particles and concentrates solutes, causing the soil Ψ_m_ and Ψ_s_ to become more negative [22]. To illustrate, while well-watered soil typically maintains a Ψ_w_ at around −0.03 MPa, severe drought can drop this value to −1.5 MPa or lower [24]. When the soil Ψ_w_ falls below that of the root cells (e.g., <−0.5 MPa), the gradient is reversed, and water flows out of the cells to reach equilibrum with the soil [25]. This results in turgor loss and a subsequent decrease in root cell turgor pressure (Ψ_p_), severely disrupting the plant’s capacity for water uptake [26].

Nevertheless, plants rapidly sense changes in hydraulic signals by detecting alterations in the osmotic environment or mechanical forces exerted on the plasma membrane or cell wall [20,27]. Key mechanosensors include mechanosensitive channels of small/large conductance (MSL) and Ca^2+^-permeable ion channels, such as Mid-Complementing Activity 1 and 2 (MCA1 and MCA2) [28,29,30,31]. Additionally, several receptor-like kinases (RLKs), e.g., Wall-Associated Kinases (WAKs), are associated with the cell wall and act as tension sensors [20,27,32]. Proteins from the OsmoSensor-related CAlcium permeable channel (OSCA) family, which function as Ca^2+^-permeable channels, as well as Histidine Kinases (HKs), seem to be crucial osmosensors under water stress conditions [33,34,35]. Local signals from these sensors converge, resulting in a rapid increase (within ~5 s) in cytosolic Ca^2+^ concentration [35]. This increase converts the mechanical signal into a biological response.

Calcium-Dependent Protein Kinases (CDPKs) regulate many downstream pathways. One of their key functions is the initiation of ROS (primarily H_2_O_2_) production in the apoplast, achieved by direct phosphorylation and activation of plasma membrane-localized Respiratory Burst Oxidase Homologs (RBOHs), such as RBOHD and RBOHF [17,36]. The generation, propagation, and neutralization of ROS by antioxidant enzymes under drought conditions together form the specific ROS signature related to the water stress [37,38,39]. One of the modes of ROS signaling involves stimulating the activation of Ca^2+^-influx channels, leading to further increase in cytosolic Ca^2+^ concentration, thus creating a positive feedback loop [40,41]. ROS and Ca^2+^, acting as secondary messengers, swiftly activate downstream protein kinases, ultimately leading to the phosphorylation of transcription factors that regulate the expression of genes critical for the drought response [42,43,44].

### 2.2. Propagation of Rapid Systemic Signals—Communication Between the Root and the Shoot

**Figure 1:**
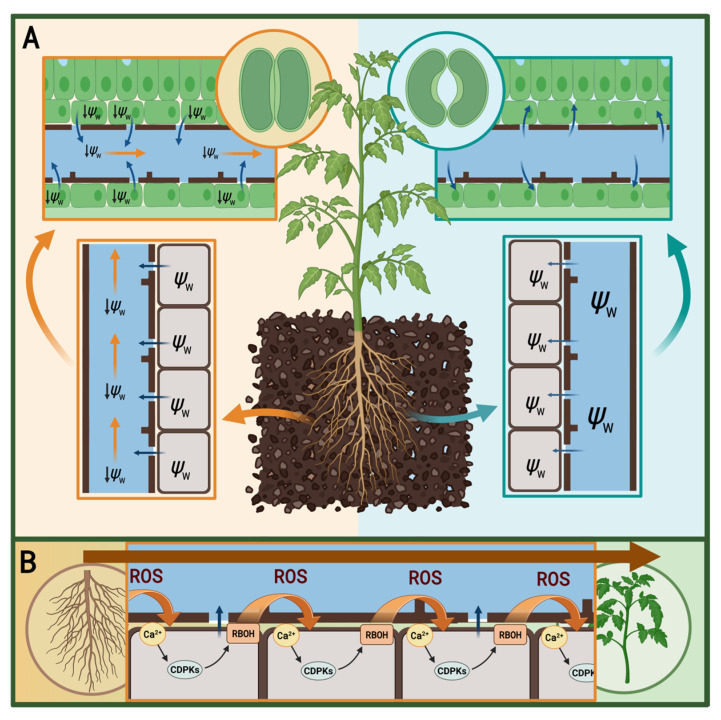
Drought warning signaling—communication from roots to shoots through hydraulic and ROS/Ca^2+^ waves. (**A**) Hydraulic waves propagating through the xylem vessels during drought (left) and normal conditions (right). (**B**) Subsequent waves of ROS and Ca^2+^ transduced via xylem under drought conditions. Created in BioRender. Michalak, A. (2025) https://BioRender.com/kn2xbjn. Accessed on 29 November 2025.

The immediate local drought response rapidly triggers a whole-plant response [17,18]. Systemic communication regarding water stress is orchestrated by fast-moving signals, including hydraulic and Ca^2+^/ROS waves, which travel acropetally from the stressed roots to the shoot [16,20,45]. Among them, the hydraulic wave is one of the first long-distance signals, enabling rapid communication about an impending drought within minutes, depending on plant height (>40 cm/min) [20,45]. The decrease in Ψ_w_ in root cells increases the tension (negative pressure) in the water column within xylem vessels. This wave of increased tension propagates rapidly up the xylem vessels toward the shoot, leading to a turgor loss in the parenchyma cells along its path (Figure 1A). This physical signal is subsequently translated by mechano- and osmosensors, and acts as a trigger for chemical signaling events, initiating a rapid oxidative burst [20]. Therefore, the hydraulic acropetal signal is immediately followed by Ca^2+^ waves and ROS signaling, triggered in cells along its path, which provide continuous information about water stress to the entire plant (Figure 1B) [16]. Upon drought sensing, locally produced ROS are released into the apoplast, where they can spread through the extracellular space. These ROS then act as triggers for Ca^2+^ release in distant cells by binding to the Hydrogen Peroxide-Induced Ca^2+^ Rises 1 (HPCA1) receptor [46]. HPCA1-mediated Ca^2+^ influx creates an acropetally traveling Ca^2+^/ROS feedback loop in the xylem and phloem of parenchyma cells [47]. The signaling waves are essential for the spatial and temporal coordination of defense responses throughout the whole plant.

### 2.3. Phytohormonal Regulation of Drought Signaling

Crucially, signal transduction pathways lead to rapid and substantial upregulation of genes encoding biosynthetic enzymes of abscisic acid (ABA), a key phytohormone involved in the drought stress response [16,48]. Among them, the 9-*cis*-epoxycarotenoid dioxygenase (NCED) family plays a pivotal role in regulating ABA biosynthesis following dehydration, as it catalyzes the rate-limiting step in this process [49]. Notably, expression of *NCED* genes, particularly *NCED3*, increases significantly and rapidly in response to environmental stresses including drought [50]. Under drought conditions, the role of ABA in shoots is extremely important, as it triggers stomatal closure, which is crucial for preventing further water loss [16,51]. Furthermore, ABA synthesized in the leaves can be transported basipetally through the phloem to the roots, where it accumulates or is subsequently recirculated via the xylem [52], playing an essential role in the plant’s systemic long-distance signaling under water stress [52,53,54]. For this reason, the involvement of ABA in modulating root architecture upon water deficit remains of paramount importance [54].

After synthesis, ABA binds to its receptors (PYR/PYL/RCARs) and triggers the canonical ABA signaling pathway [55]. Subsequently, the ABA-receptor complex inhibits the negative regulatory Protein Phosphatases 2C Clade A (PP2C.A) [56,57]. This leads to the release from inhibition of the central positive regulators of the signaling pathway—the protein kinases known as SnRK2s (specifically OST1/SnRK2.6), enabling their activation and initiating downstream phosphorylation events [58,59]. SnRK2s interact with two primary categories of target proteins: membrane transporters/channels and nuclear transcription factors [60]. In guard cells, SnRK2s quickly phosphorylate and activate plasma membrane ion channels, initiating stomatal closure fundamental for preventing further water loss [61,62,63]. In addition, the activated SnRK2s translocate to the nucleus, where they phosphorylate specific transcription factors, controlling the expression of drought stress-responsive genes [64,65].

Recent studies have identified small peptides, including CLvata3/Embryo-surrounding region-related 25 and 9 (CLE25 and CLE9), as chemical messengers participating in the water deficiency response [66,67,68,69]. Under drought conditions, the gene encoding CLE25 is upregulated in root tissues, and the peptide is transported to the aerial parts of the plant [66]. Although CLE25 is known to move acropetally from roots to shoots, the exact mechanism of its transport remains unknown. Current models suggest that it may be loaded into xylem or phloem for long-distance delivery [70]. In leaf cells, CLE25 is perceived by BAM1 and BAM3 receptor-like kinases, and its binding initiates an intracellular signaling cascade that upregulates genes encoding the ABA biosynthetic enzyme NCED3. By activating ABA synthesis in the shoot, CLE25 reinforces translation of locally perceived stress signal in roots into the ultimate water-saving response—the ABA-mediated stomatal closure [66]. Another CLE peptide, CLE9, has also been identified as a key element of the ABA-dependent drought response [69]. CLE9 promotes stomatal closure by triggering the activation of anion efflux channels in the plasma membrane of guard cells, supported by the stimulation of H_2_O_2_ and nitric oxide (NO) production [69].

While ABA is the master regulator of plant responses to drought, it relies on a complex network of hormonal crosstalk [14,16,71]. To balance competing demands, such as water conservation versus photosynthesis, ABA interacts synergistically or antagonistically with other phytohormones, including auxins, brassinosteroids, cytokinins, jasmonic acid, melatonin, and strigolactones [19,72]. These key interactions are summarized in Table 1.

While the hormonal interactions detailed above highlight ABA’s central role, drought response strategies vary significantly across species [88]. Plants are generally categorized as isohydric or anisohydric. Isohydric plants maintain stable leaf water potential through rapid, ABA-driven stomatal closure, whereas anisohydric plants maintain partial stomatal opening to sustain photosynthesis, thereby tolerating significantly lower leaf water potentials [89]. For instance, in economically valuable anisohydric crops such as grapevine or wheat, early stomatal closure is often triggered primarily by hydraulic signals rather than ABA accumulation alone [51,90]. Crucially, these behaviors do not represent a rigid dichotomy but rather a continuous spectrum of stomatal regulation within one species, depending on its cultivar, as well as the intensity and duration of water stress conditions [91,92].

## 3. Key Transcription Factors in Drought Response

Perceived drought signals, described above, induce rapid and highly coordinated transcriptional changes in plants. They are mediated by TFs, proteins that bind to specific cis-acting elements of DNA, such as promotors and enhancers, thereby regulating gene expression [93]. Although plants possess a large number of TF families (estimated to be at least 56) [94,95], only some of them are predominantly implicated in response to drought stress, e.g., NAC (NAM, ATAF1/2, and CUC2), AP2/ERF (APETALA2/Ethylene Responsive Factor), WRKY, bZIP (basic region/leucine zipper), MYB (v-myb avian myeloblastosis viral oncogene homolog), HD-ZIP (Homeodomain-Leucine Zipper), ZnF (zinc finger), bHLH (basic helix-loop-helix), ASR (abscisic acid, stress, ripening induced), NF-Y (Nuclear factor Y) and HSF (Heat shock factor) [95]. Numerous studies over the years highlight that TFs, acting as either positive or negative regulators, form regulatory networks specific to individual plants [95]. Representative TFs of Arabidopsis, rice, corn, wheat, tomato, and grapevine, as well as their functions under drought stress are listed in Table 2. Consequently, this poses a significant challenge in defining a single universal transcriptional response to drought stress in diverse plant species. Despite this complexity, the overall regulatory mechanisms governing TF networks are now well characterized.

### 3.1. ABA-Dependent Pathway

**Figure 2:**
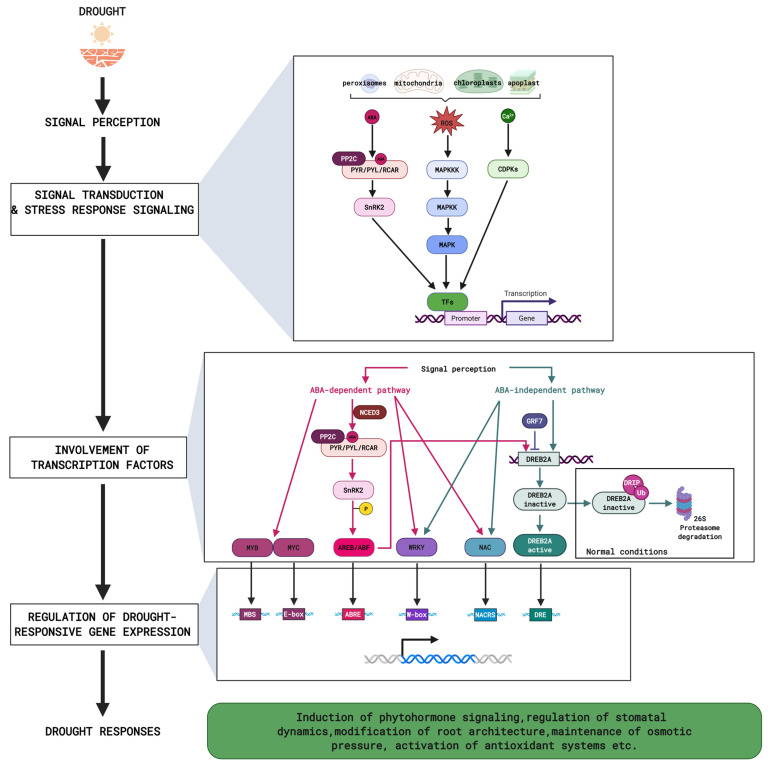
Main transcription factors regulating drought-induced signaling pathways. Created in BioRender. Michalak, A. (2025) https://BioRender.com/c3iv8ch. Accessed on 23 December 2025.

TF-mediated plant response to drought stress is primarily regulated by two interconnected pathways: ABA-dependent and ABA-independent (Figure 2) [160]. In the ABA-dependent route, the most common mechanism of activation involves the phosphorylation of proteins by the SnRK2 kinases [160]. This process is particularly well established in the case of ABRE (ABA-Responsive Elements)-binding protein/ABRE-binding factors (AREB/ABF) transcription factors, which belong to the bZIP family [65,161]. They are master TFs in drought-dependent ABA signaling. Once phosphorylated by SnRK2, they can bind to the ABRE sequence located in the promoter region of target genes, thereby activating their expression (Figure 2) [118]. Although AREB/ABFs play a key role, other TF families are also crucial contributors in the ABA-dependent response of plants to drought stress. MYB family participates in plant responses to various abiotic stresses by binding to the MBS (MYB-binding sites) of their target genes [162]. Most of the MYB transcription factors, responding to drought stress, belong to the R2R3-MYB subfamily and regulate, hormone signaling, antioxidant defense, osmoprotectant biosynthesis, and morphological changes [95,96]. These TFs usually cooperate with AREB/ABF together with the MYC subfamily, belonging to the bHLH family, which plays a key role in JA signaling induced in plants under drought conditions (Figure 2) [118]. For example, in Arabidopsis guard cells, beside ABRE and MBS cis-acting elements, a number of ABA-responsive genes share a MYC2 binding site called E-box [163], which emphasize that the precise regulation of stomatal opening and closing requires the cooperation of multiple TFs.

### 3.2. ABA-Independent Pathway

The most extensively studied and characterized transcription factors, governing ABA-independent response, are members of the DREB (Drought Responsive Element-Binding) subfamily, which belong to the AP2/ERF superfamily [119]. They recognize the cis-acting element DRE/CRT to induce the expression of their downstream genes (Figure 2) [164]. Among the DREB TFs, DREB2 from the A2 subgroup is mainly involved in plant reactions to dehydration and osmotic stress. Unlike bZIP transcription factors involved in ABA-dependent signaling and activated through phosphorylation, expression and induction of the DREB2A are tightly controlled under normal conditions due to their adverse effects on plant growth [128]. At the transcriptional level, their expression is managed by a GRF7 (Growth-Regulating Factor 7) transcription factor acting as a repressor [165]. At the protein level, DREB2A is ubiquitinated by ubiquitin E3 ligases DRIP (DREB2-Interacting Protein) and degraded through the 26S proteasome pathway (Figure 2) [166]. Interestingly, *DREB2A* gene also contains the ABRE sequence. Studies conducted by Kim et al. [167] in Arabidopsis showed that both osmotic stress and AREB TFs are necessary to induce its expression. This highlights the crosstalk between different transcription factors, as well as between ABA-dependent and ABA-independent pathways.

### 3.3. Integration of ABA-Dependent and ABA-Independent Signals

In addition to AREB, the NAC and WRKY families serve as good examples of transcription factors that integrate signals from both pathways (Figure 2) [168,169,170]. WRKYs can act as both positive and negative regulators of gene expression. They bind to the W-box sequence in their target genes [170]. WRKYs not only directly regulate gene transcription but also indirectly affect the plant response to drought by interacting with other proteins. Moreover, WRKY promotors harbor ABRE, DRE and G-box cis-acting elements. This indicates that some TFs themselves are regulated by both ABA-dependent and ABA-independent pathways [170,171].

Similarly to WRKY, NAC transcription factors also function as positive or negative regulators in plant reactions to drought stress. They bind to the NACRS (NAC-recognition sequence) element in their downstream target genes but can also interact with other transcription factors [124,172]. Expression of *NAC* genes, containing ABRE and DRE cis-acting elements, as well as JA and SA responsive elements is activated in both ABA-dependent and ABA-independent pathways [172]. Additionally, NACs undergo post-transcriptional modifications, such as miRNA-mediated degradation and alternative splicing, as well as post-translational phosphorylation, ubiquitination, proteolysis or dimerization, which fine-tunes plant response to water scarcity conditions [124,172,173,174].

The extensive network of transcription factors responding to drought stress remains to be fully elucidated. This is further complicated by the diverse mechanisms governing functioning of different plants under water deficient conditions, which makes it impossible to simply extrapolate the results obtained from one, even model species to another [95].

The role of transcription factors responding to drought stress in crop plants has been described in detail in many available comprehensive reviews [99,108,113,122]. Furthermore, in most cases, the transcription factors discussed rarely regulate a single gene or a single stress response mechanism. Much more frequently, they simultaneously control the expression of many target genes, which themselves are involved in diverse but interrelated cellular mechanisms/processes [128]. Those include ABA biosynthesis, signaling of other phytohormones, such as BRs and JA, stomatal dynamics, root architecture, osmotic pressure maintenance, or antioxidant defense [128].

## 4. Accumulation of Osmolytes and Cellular Protectants

Abiotic stresses, including drought, induce excessive ROS accumulation, resulting in oxidative protein degradation and lipid peroxidation [175]. Under drought stress, plants activate several protective physiological and biochemical mechanisms to adapt to the changing conditions. One of them is the production of osmoprotectants, i.e., low-molecular weight, compatible solutes that are electrically neutral and non-toxic [176]. Once accumulated inside the cell, they play a dual function. On the one hand, they provide cytoprotection by stabilizing cellular proteins, enzymes, and membranes. On the other hand, they enable osmoregulation by lowering the cellular osmotic potential to facilitate turgor maintenance [176,177]. Plant osmoprotectants can be broadly categorized into four groups: ammonium compounds, sugars, sugar alcohols (polyols), and amino acids [176].

### 4.1. Ammonium Compounds

**Figure 3:**
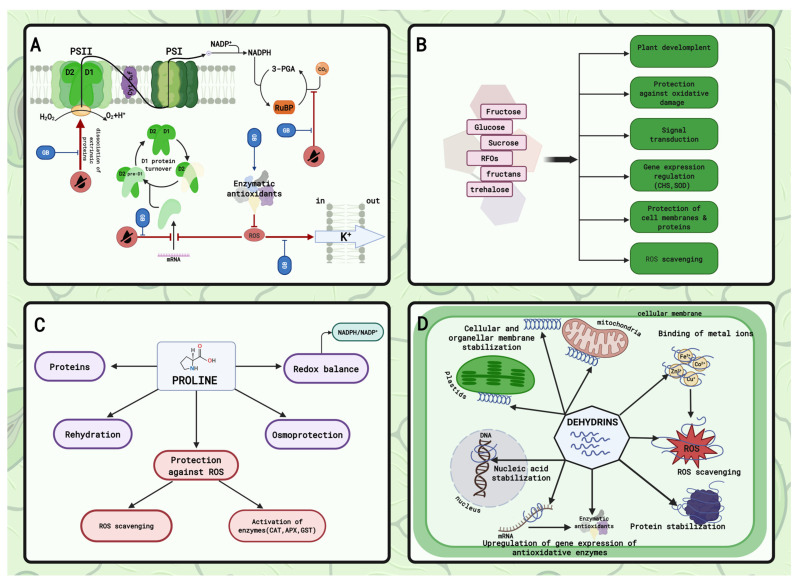
Osmolytes and cellular protectants functioning in plant cells under drought stress. (**A**) Role of glycine betaine; (**B**) sugars and polyols; (**C**) proline; (**D**) dehydrins in drought stress. Created in BioRender. Michalak, A. (2025) https://BioRender.com/sde8vdq. Accessed on 23 December 2025.

One of the most frequently described and studied groups of osmolytes are ammonium compounds, including betaines, especially glycine betaine (GB) (Figure 3A) and β-alanine betaine [176]. GB is a quaternary ammonium cation, which accumulates in tissues to an extent depending on the plant stress tolerance [178]. It is a key compatible solute synthesized in chloroplasts, where it is involved in maintaining photosynthetic efficiency by protecting thylakoid membranes, enzymes, and protein complexes, including Photosystem II (PSII) (Figure 3A). On the other hand, it can also be translocated to cytoplasm [179,180]. The role of GB in mitigating harmful effects of ROS production during drought stress is multifaceted. Glycine betaine is able to stabilize the structure of PSII by decreasing the dissociation capacity of extrinsic proteins and facilitating the repair and turnover of D1 protein [179]. Although GB is not generally considered to be an effective free radical scavenger, it can activate and stabilize the activity of antioxidant enzymes (Figure 3A) [176]. Finally, it helps to maintain CO_2_ fixation by protecting RiBulose-1,5-BIS-phosphate Carboxylase/Oxygenase (Rubisco) and Rubisco activase [181] and limits the ROS-induced efflux of K^+^ ions (Figure 3A) [179,181].

### 4.2. Sugars and Polyols

Under drought stress, plants accumulate sugars and polyols (Figure 3B) [12]. Contrary to GB, sugars act as the main osmoprotectants for osmotic adjustment [182]. The precise functions of sugars under abiotic stresses vary, depending on their properties. Under oxidative stress caused by water scarcity, glucose and fructose play a key role in coordination of plant development [183]. Additionally, since glucose is the initial precursor in the biosynthesis of carotenoids, ascorbate, as well as glutathione-building amino acids, it contributes to protection against oxidative damage caused by ROS [184]. Both glucose and sucrose are also involved in signal transduction and regulation of gene expression related to chalcone synthase or SOD [184,185]. Another important group of carbohydrates, SOS (Sucrosyl OligoSaccharides), include sugars derived from sucrose, such as raffinose family of oligosaccharides (RFOs) and fructans [183]. Raffinose, synthesized in cytoplasm, and fructans produced in vacuole but also detected in apoplast, can protect cell membranes by interacting with them under drought stress. They can also act directly or indirectly as antioxidative molecules [183,186]. Trehalose, a non-reducing and non-reactive sugar, is a stress protector functioning in ROS scavenging, gene expression modulation, and signaling pathways, mainly associated with plant response to dehydration [178]. It also protects membranes and proteins through interaction with their amorphous glassy structure (Figure 3B) [178,187].

Polyols can be grouped into two categories based on their structure: cyclic (myoinositol, pinitol) and linear (mannitol, sorbitol). Mannitol and inositol are two of the most often described polyols in the context of plant reactions to abiotic stresses. Of these two substances, mannitol is the most common and most frequently accumulated osmoprotectant, while different isomers of inositol act as additional osmolytes [178,188]. As osmolytes, polyols assist in water retention in cytoplasm and in sodium sequestration into the vacuoles and apoplast. Polyols can also function as cytoprotectants by interacting with proteins, and cellular membranes, as well as chaperones and ROS scavengers [189].

### 4.3. Amino Acids

The fourth group of osmoprotectants are amino acids. Many of them, including proline, alanine, arginine, glycine, serine, leucine, valine, glutamine, asparagine, citrulline, ornithine, and γ-Aminobutyric acid (GABA), accumulate in plant cells in response to abiotic stresses [178,189]. Among them, proline seems to be particularly important under drought stress conditions (Figure 3C) [12]. Under water deficit, the proline content increases mainly in the cytoplasm and chloroplasts [190] as a result of enhanced biosynthesis or/and inhibited degradation. It is still unclear, however, whether this is a sign of stress or a stress response [178]. In plant cells, proline participates in many stress-related processes including osmoregulation, antioxidant defense—directly by ROS scavenging and indirectly by activating detoxification, and maintaining low NADPH to NADP^+^ ratio (Figure 3C) [12,182].

### 4.4. Dehydrins—Cellular Protectants

Dehydrins belong to the group II of Late Embryogenesis Abundant (LEA) protein family. They are widely distributed molecules that adopt their defined secondary structure only when bound to a ligand like a protein, membrane, DNA, or metal ion (Figure 3D) [191,192,193]. A characteristic feature of dehydrins is the lack of stable secondary and tertiary structure (i.e., they are Intrinsically Disordered Proteins—IDPs). This means that they do not undergo denaturation even under stress conditions that usually induce protein damage. The function performed by dehydrins is variable—a feature defining them as moonlighting proteins—because it is related to their sequence motif, localization, and conditions inside the cell [191,194]. Dehydrin expression at both gene and protein levels is upregulated under drought stress [195]. Their interaction with phospholipids stabilizes and protects cellular and organellar membranes. Similarly, they are able to stabilize and prevent protein aggregation, as well as protect DNA from damage. To avoid oxidative damage, they bind to metal ions, such as Fe^3+^, Zn^2+^, Cu^+^, Co^2+^, responsible for ROS formation. Moreover, they upregulate gene expression of antioxidative enzymes and additionally function as ROS scavenges (Figure 3D) [191,193,196].

### 4.5. Potassium Ions

Among the various osmolytes, potassium ions (K^+^) play a paramount role as the primary inorganic ions responsible for cellular osmotic adjustment [197]. Under drought conditions, plants actively increase uptake of K^+^ from the soil solution via high-affinity root transporters (e.g., HAK5) and channels (e.g., AKT1) [198]. Once inside the cell, K^+^ is sequestered into the vacuole via tonoplast antiporters (NHX) to significantly reduce the cellular osmotic potential [199,200]. This reduction in osmotic potential maintains cellular turgor, as well as the water potential gradient between the root and the soil. This is necessary for continuous water uptake, even when soil moisture levels decline [201,202]. Osmotic adjustment through the regulation of intracellular K^+^ concentration is also essential for sustaining the turgor pressure required for cell expansion and stomatal regulation during the water stress response [203]. Furthermore, under low water-regime, optimal K^+^ level minimizes ROS production and oxidative stress-related damage, improving antioxidant status of the cells. Potassium has also been found to increase the content of other osmoprotectants. K^+^ application has been shown to improve the concentration of soluble sugars and free amino acids, involved in drought stress tolerance [204,205]. For this reason, understanding the molecular mechanisms and key regulators responsible for maintaining potassium homeostasis in plants is essential for modifying plants to develop traits that enable water retention and drought survival [197].

## 5. ROS Generation and Effects of Oxidative Stress

Generation of ROS is an unavoidable part of aerobic metabolism [206]. Atmospheric oxygen, due to its configuration in its ground state (triplet oxygen, ^3^O_2_), has a restricted ability to react with cellular and organelle structures [207]. In cells, oxygen can be partially reduced/activated through biochemical reactions, electron transport chains (ETCs), ultraviolet-B (UV-B) light, and ionizing radiation, leading to formation of ROS [207]. ROS have strong oxidative potential and high reactivity towards cellular macromolecules [208]. They are classified into two main forms: free radicals, such as O_2_^•−^ (superoxide radical), ^•^OH (hydroxyl radical) or HO_2_^•^ (hydroperoxyl radical), and non-radicals, such as H_2_O_2_ (hydrogen peroxide) and ^1^O_2_ (singlet oxygen) [44].

### 5.1. Sites of ROS Production in Plants

**Figure 4:**
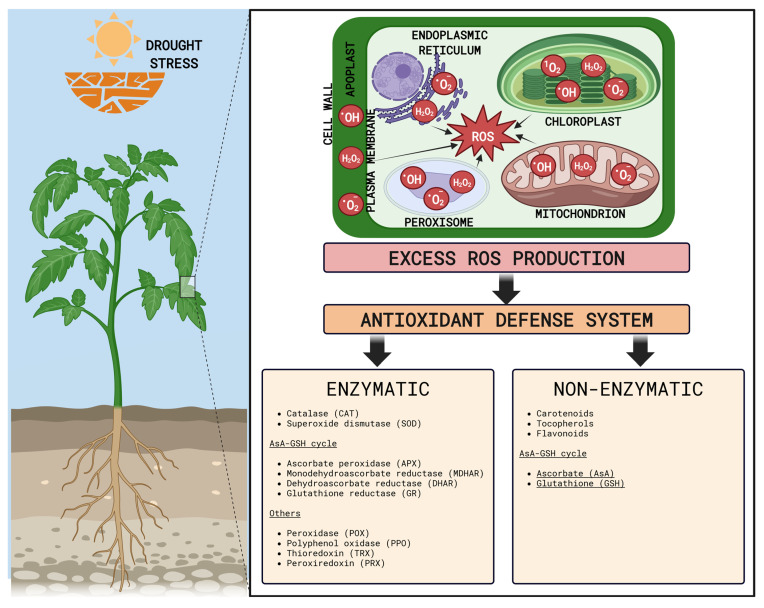
Sites of ROS generation and an overview of antioxidant defense systems in plant cells. Created in BioRender. Michalak, A. (2025) https://BioRender.com/btzcnee. Accessed on 29 November 2025.

Chloroplasts are the major site of ROS production in green plant tissues, and a unique site of constitutive formation of singlet oxygen (Figure 4) [209]. There are two predominant sources of increased ROS production in thylakoids: triplet chlorophyll (^3^Chl*) in PSII, which produces ^1^O_2_ through a reaction with ^3^O_2_, and over-reduced electron transport chain (chlETC), in which electrons are transferred directly to O_2_, generating O_2_^•−^ [210,211]. In the latter, the formation of ROS occurs mainly due to electron leakage from ferredoxin (Fd) via the Mehler reaction, but they can also be generated within PSII or at plastoquinone (PQ) pool. In the next step, O_2_^•−^ is disproportionated by SOD (Superoxide dismutase) to H_2_O_2_, which in turn can be used in Fenton reaction to produce OH^•^ [175]. Under drought stress, stomatal closure lowers the availability of intracellular CO_2_ and is the main reason of ETC overreduction [212].

Contrary to green tissues, in roots, mitochondria are the main site of ROS production (Figure 4) [39,213]. Similarly to chloroplasts, the generation of O_2_^•−^ is mostly due to the electron leakage from ETC (mitochondrial ETC, mtETC). In the case of plant mitochondria under drought stress, increased ROS generation occurs at Complex I (NADH dehydrogenase) and Complex III (Cyt c reductase). Subsequently, as a result of the reaction catalyzed by SOD, O_2_^•−^ is converted to H_2_O_2_ [39,213]. Mitochondrial alternative oxidase (AOX1), which is induced by ROS, is able to lower ROS levels by keeping the ubiquinone (UQ) pool in its reduced state. Interestingly, plants lacking this enzyme are more sensitive to drought stress [214].

Peroxisomes are another site of ROS generation and owe their name to hydrogen peroxide which accumulates within them in large quantities (Figure 4). H_2_O_2_ production is mainly the result of Glycolate oxidase (GOX) function, which increases during drought stress [212]. It is also produced by β-oxidation of fatty acids, SOD, Polyamine oxidase (PAO), Copper amine oxidase (CuAOs), Sulfite oxidase (SO), and Sarcosine oxidase (SOX) [212]. Peroxisomes, similarly to chloroplast and mitochondria, possess a short ETC consisting of NADH and Cyt b, which, together with Xanthine oxidase (XO), is the source of O_2_^•−^ for these organelles. During drought stress, when photorespiration is triggered, peroxisomes become the main place of H_2_O_2_ production [212,215].

Interestingly, endoplasmic reticulum (ER) is also a site of ROS generation (Figure 4) [209]. On the one hand, this is related to the activity of ER oxidoreductase (ERO), producing H_2_O_2_ necessary for proper protein folding, and on the other hand, to the O_2_^•−^ formation due to the oxidation and hydroxylation processes at P450 cytochrome reductase [209]. Under water scarcity conditions, ER-derived ROS are involved in triggering stress-induced programed cell death [216].

The apoplast participates in the generation of extracellular ROS mainly by the activity of plasma membrane NADPH oxidases (RBOHs), and cell wall Peroxidases (POXs) (Figure 4) [217]. Together with other enzymes, such as Quinine reductases, Lipoxygenases (LOXs), and CuAOs, they are responsible for the creation of oxidative burst. Nonetheless, the resulting H_2_O_2_ and O_2_^•−^ play an important role in signaling and cell wall modifications under stress conditions, including drought [207,218].

### 5.2. ROS Signaling and Antioxidant Systems

As described above, ROS are generated constitutively in plant cells, and during evolution, these organisms developed several mechanisms to use them as signaling molecules. Each cellular compartment exhibits its own ROS signature that can change due to various stress conditions. Consequently, plants must decipher such signatures to initiate the appropriate, stress-specific signal [39]. These signals can be classified as external (apoplastic), internal (cytosolic and nuclear), and organellar (mitochondrial, chloroplastic, and peroxisomal) [208]. Unlike O_2_^•−^, OH^•^, and ^1^O_2_ with their short half-lives, H_2_O_2_ has a relatively long half-life and is diffusible. This allows it to function as a redox signaling molecule and be readily transported across membranes [217,219]. The transport of hydrogen peroxide may also be mediated by aquaporins. In Arabidopsis, they include tonoplast intrinsic proteins (TIPs), such as AtTIP1;1 and AtTIPTIP1;2 [220], as well as plasma membrane intrinsic proteins from subfamily 2 (PIP2), such as PIP2;1, PIP2;2, PIP2;4, PIP2;5, PIP2,7 [221]. As a signaling molecule, H_2_O_2_ can travel between organelles and even cells, and interact with proteins, oxidizing them. This can lead to activation of transcription factors, for example, belonging to NAC and WRKY families, as well as calmodulin-binding transcription activators [222]. Additionally, in plant cells, multiple cascades require combined Ca^2+^ and H_2_O_2_-dependent signals [222]. For example, during a ROS wave, a drought stress triggers calcium influx to the cell cytosol, which in turn either activates RBOHs or leads to the activation of CDPKs responsible for phosphorylation and activation of RBOHs. Apoplast-generated ROS can then be sensed by neighboring cells [39,223,224,225]. Drought stress-related ROS accumulation in cells, beside calcium-dependent pathways, also triggers the MAPK cascade necessary for signal transduction within the cells [44].

The balance between the generation and neutralization of ROS by various antioxidant systems is called redox homeostasis [207]. While a basal level of ROS is necessary for redox signaling, any disturbances in this equilibrium, mainly caused by ROS overproduction, lead to oxidative stress due to ROS overaccumulation. This results in serious detrimental effects on proteins, membranes, nucleic acids, and carbohydrates, leading to cellular damage, disruption of physiological processes, and even cell death [207,208]. To protect themselves, plants activate antioxidant defense systems consisting of antioxidant enzymes and non-enzymatic antioxidants (Figure 4).

#### 5.2.1. Enzymatic Antioxidant System

There are several antioxidant enzymes functioning in plant cells (Figure 4) [207]. SOD activity is considered to be the primary defense mechanism against ROS. By dismutating O_2_^•−^ into H_2_O_2_, it prevents the formation of highly reactive ^•^OH [226]. Three main types of SOD, classified according to their metal cofactors, have been identified in plant cells. They include Cu/Zn-SOD localized in chloroplasts and cytosol, chloroplastic Fe-SOD, and mitochondrial Mn-SOD [226]. The resulting H_2_O_2_ can then be decomposed by catalase (CAT), a heme-containing enzyme present in all aerobic organisms [207]. In plants, it occurs primarily in peroxisomes, but also in mitochondria and cytosol. This protein is able to convert 26 million H_2_O_2_ molecules into H_2_O in just one minute [207].

Next set of enzymes is part of the major antioxidant defense system called the Asada-Halliwell cycle or ascorbate-glutathione (AsA-GSH) pathway, which links both enzymatic and non-enzymatic antioxidants. This cycle was found in many cellular compartments, including cytosol, mitochondria, chloroplasts, peroxisomes, and glyoxysomes. As the first step, Ascorbate peroxidase (APX) scavenges H_2_O_2_ and simultaneously oxidizes AsA, producing monodehydroascorbate (MDHA), which can further disproportionate to (DHA) [208]. AsA regeneration is catalyzed either by the NADH/NADPH-dependent enzyme—Monodehydroascorbate reductase (MDHAR) using MDHA as a substrate or by the GSH-dependent Dehydroascorbate reductase (DHAR) using DHA. In this reaction, DHAR utilizes reduced glutathione (GSH), producing its oxidized form (GSSG). In plant cells, the recycling of DHA can also happen spontaneously, without enzyme assistance, but the rate of the reaction is inferior to that of DHAR [207,208]. Finally, Glutathione reductase (GR) regenerates GSH by reducing GSSG in a NADPH-dependent reaction, which is essential to maintain redox homeostasis [207]. Other antioxidant enzymes include POX, Polyphenol oxidase (PPO), Thioredoxins (TRX), and Peroxiredoxins (PRX) [207].

Many studies over the years focused on the effects of drought, gene expression, and activity of enzymatic antioxidants in the context of both varied water conditions and different plant drought tolerance. The collected data showed that there is no universal and consistent plant response to drought stress in terms of ROS scavenging [212,227]. While increased activity of individual enzymes was observed in some plant species and varieties, no changes were found in others [228,229,230,231].

#### 5.2.2. Non-Enzymatic Antioxidant System

Non-enzymatic antioxidants can be divided into two groups: lipophilic (tocopherols, carotenoids) and water-soluble (AsA, GSH) (Figure 4) [175]. As mentioned above, ascorbate and glutathione play a pivotal role in the AsA-GSH patway. Ascorbic acid (vitamin C), due to its ability to donate electrons, plays a significant role in ROS scavenging by plants. Primarily synthetized in mitochondria, it is then delivered to other compartments either via facilitated diffusion or proton gradient-dependent transport [175]. Besides its role in the AsA-GSH pathway, it can also directly remove free oxygen radicals and regenerate tocopherol (Vitamin E) from the tocopheroxyl radical [207,208]. Moreover, under drought stress, AsA plays an important role in growth and development of plants, regulating the cellular water status and biosynthetic pathways of many phytohormones [207,232]. GSH, in addition to its function in AsA regeneration, directly scavenges ROS. Glutathione is also responsible for inducing various defense mechanisms through redox signaling, making it a key element of signaling cascades activated in plant cells during abiotic stress [207,208].

Tocopherols are chloroplastic antioxidants that maintain photosynthesis by scavenging ROS, especially singlet oxygen and hydroxyl radical. They accumulate during drought stress, thus protecting photosynthetic machinery from both photo-oxidation and auto-oxidation of lipids in chloroplast membranes [208,233]. The role of carotenoids, like that of tocopherols, is closely related to the protection of photosynthetic apparatus [234]. As a part of photosynthetic antennas, they can reduce stress symptoms caused by intensive light. During photosynthesis, they are also able to stabilize thylakoid membranes and protect chlETC proteins by scavenging several different radicals, such as peroxyl (ROO^•^), ^•^OH or O^2−•^, as well as eliminating ^1^Chl^*^, ^3^Chl^*^ and ^1^O_2_ [207,208,234]. Furthermore, flavonoids, especially flavones and flavonols, have the ability to scavenge free radicals in plants cells. Their function is mainly associated with protection against lipid peroxidation [207,235].

### 5.3. Methods for Studying Oxidative Stress in Plants

Studying ROS production and oxidative stress, occurring in plants during drought requires a multifaceted approach. The choice of appropriate methods depends largely on the working hypothesis, the types of plant material used, and the selected plant species, since not all techniques are universally applicable. Each detection method also has its own advantages and disadvantages [236]. An additional difficulty is the detection and quantification of ROS produced in cells which is challenging due to their specific properties, such as short lifetime, instability, potential for mutual interaction, and different sites of production [236]. The methods employed can therefore be divided into direct and indirect, qualitative and quantitative, or applicable in vitro or in vivo, and are listed in Table 3 [219,236,237,238,239].

#### 5.3.1. Indirect Methods

Indirect methods concentrate on measuring the effects of ROS activity and subsequent oxidative stress. One approach is based on biochemical estimation of plant pigments, malondialdehyde (MDA), a product of lipid peroxidation, and osmolytes [239]. The second approach focuses on various assays measuring the levels of activity of antioxidative enzymes and accumulation of non-enzymatic antioxidants [239]. Most of these methods involve spectrophotometric measurements at specific wavelengths. They require sample preparation through homogenization and centrifugation, and the measurements performed are taken in vitro using the obtained supernatant. Although relatively simple, fast, and cost effective, they are typically characterized by relatively low sensitivity and are inapplicable to in vivo systems [236,239]. They are routinely used in many drought stress studies to assess the impact of water availability or to compare drought-sensitive and tolerant species [228,229,230,231]. Another popular approach to indirect in vitro measurements is chromatography, particularly High-Performance Liquid Chromatography (HPLC). Unlike UV-Vis measurements, it is highly specific and sensitive, with a downside of requiring complex instrumentation and time-consuming sample preparation [236,239]. HPLC can be used to assess the accumulation of osmolytes under drought stress [240,241,242,243].

#### 5.3.2. Direct Methods

Direct methods enable researchers to measure changes in ROS levels not only in cells, but also in specific organelles. These approaches use genetically encoded or biochemical probes and biosensors. With constant advancement in this field, sensors are regularly improved and developed [238]. Probes are usually membrane-permeable and are applied in their reduced (colorless or nonfluorescent) form. Upon entering the cell or organelle, they become oxidized, which results in color or fluorescence changes [238].

One of the most popular colorimetric probes used for in situ ROS detection is 3,3′diaminobenzidine (DAB) and Nitroblue tetrazolium (NBT). Unfortunately, these relatively simple-to-use histochemical techniques come with a lot of disadvantages. Although they can provide qualitative (NBT) or/and semi-quantitative (DAB) results, they are characterized by low sensitivity, limited specificity, and harsh protocols [219,236,238]. Nevertheless, both techniques are routinely used to assess ROS production in plants subjected to drought stress. They are used, for example, to assess drought-protective effect of plant treatments with bacteria [244,245], to study the effect of selected proteins including transcription factors on plant drought response [246,247] or, in line with the current demand for drought-tolerant species, to characterize new lines of crop plants such as rice [248] or tomato [249].

Since colorimetric-based probes are characterized by low sensitivity, a new set of probes with high sensitivity, high reactivity, and the ability to provide quantifiable results was designed [219]. Fluorescent-based approach is a non-invasive method, applied both in in vitro and in vivo studies. It is characterized by a high signal-to-noise ratio, precise detection of ROS interactions within cellular systems, including specific location and time of interaction, as well as minimal cross-reactivity with cellular antioxidants [219,237,238]. The developed probes, including CM H_2_DCF-DA (Chloromethyl 2′,7′-dichlorodihydro-fluorescein diacetate), OxyBurst Green (H_2_HFF-BSA Dihydro-2′,4,5,6,7,7′-hexafluoro-fluorescein), DHR (dihydrorhodamine 123), can detect ROS in general [236,238]. The other can be used for specific oxygen species, among them are: BESH2O2-Ac (Acetyl-6′-O-Pentafluoro-benzenesulfonyl 2′-7′-difluoro-fluorescein), Ample Rex (*N*-acetyl-3,7-dihydroxyphenoxazine, POX substrate), boronate-based probes, NBCD (*N*-borylbenzyloxycarbonyl-3,7-dihydroxyphenoxazine), DHE (dihydroethidium (2,7-diamino-10-ethyl- 9-phenyl-9,10-dihydrophenanthridine), MitoSOX (mitochondria localizing derivative of dihydroethidium), DanePy (3-[*N*-(β-diethylaminoethyl)-*N*-dansyl]aminomethyl-2,2,5,5-tetramethyl-2,5-dihydro-1*H*-pyrrole), and SOSG (Singlet Oxygen Sensor Green) [236,238]. They are constantly modified to improve their properties (including localization), as they can be non-specific and autooxidized or photobleached in cells. The use of fluorescent-based probes is well documented in studies on drought stress in Arabidopsis [238,250,251].

The genetically encoded fluorescent protein biosensors enable plant researchers to dynamically monitor ROS status in vivo during stress conditions, such as drought [238]. This approach, similarly to fluorescent-based chemical probes, is non-invasive, but unlike the previous method, it enables long-term imaging of ROS levels in tissues and organelles throughout the plant’s life cycle. Unfortunately, one of the disadvantages of biosensors is the need to perform plant transformation, a technique that is not readily available for all plant species [238]. The first applied systems were used for general ROS measurements and included roGFP1 and roGFP2 [252], as well as rxYFP sensors [253]. To overcome this limitation, the next iteration of biosensors was specific towards distinct ROS, including roGFP-Orp1 sensor created based on roGFP [254], and cpYFP sensor on rxYFP [253]. Another set of specific biosensors includes the HyPer family, sensors that are a fusion of cpYFP and *Escherichia coli* H_2_O_2_-responsive regulatory domain OxyR. Each member of the HyPer family has slightly different properties and applications [255,256,257,258]. Fluorescent protein-based genetically encoded biosensors have also been further developed for use in the whole-plant fluorescence imaging, firstly in the model plant *A. thaliana* [259,260], and more recently to study crop plants grown in soil under drought stress [261].

## 6. Mechanisms of Cell Wall Remodeling and Growth Regulation

Cell expansion, a crucial element of plant growth processes, is directly related to water uptake. According to the fundamental principles of plant physiology, cell expansion is a turgor-driven process, in which internal hydrostatic pressure (P) acts against a yielding cell wall (CW) to produce an increase in the cell volume [262,263]. Under optimal conditions, the influx of water increases the turgor necessary to exceed the mechanical yield threshold (Y) of the CW. However, under drought stress, the external soil water potential decreases, reducing the gradient for water uptake and threatening to lower cell turgor below the critical threshold required for growth [264,265]. In theory, this should result in an immediate cessation of growth. However, studies have shown that although shoot growth is often rapidly inhibited to conserve resources and reduce transpiration surface, the primary root system maintains, or even accelerates, elongation into deeper soil layers to access residual moisture [266]. This divergence in growth responses represents an active, metabolically expensive reprogramming of the CW’s plasticity/rheological properties [267]. CW is a dynamic, metabolically active extracellular matrix that undergoes remodeling in response to water deficit. This remodeling is orchestrated by a complex signaling network involving ABA, ROS, and CW integrity (CWI) sensors, which collectively modulate the activity of wall-modifying agents, such as EXPansins (EXPs), Xyloglucan endoTransglucosylases/Hydrolases (XTHs), and Pectin MEthylesterases (PMEs) [265].

### 6.1. Cell Wall Mechanics in Expansion Growth

The cell expansion mechanism is described by The Lockhart model, which includes the dependence of expansive growth (*GR*) on cell turgor and CW yielding properties:

(1) GR= ϕ(P−Y)

where *ϕ* is CW extensibility, P is turgor pressure and Y is CW’s yield threshold.

The immediate physical consequence of drought stress is a reduction in cell turgor [263,268]. If wall properties (*ϕ* and Y) remained constant, growth would stop as soon as P fell below Y. However, data indicate that plants actively modulate *ϕ* and Y to sustain growth despite reduced turgor. For instance, in the elongation zone of roots, a full recovery of expansion rate was observed even in case of incomplete restoration of turgor [269,270]. This implies the presence of compensatory regulation, in which CW becomes significantly “looser” (increased *ϕ*), or the yield threshold is lowered (Y decreases) to permit expansion at lower pressure [268]. Recent studies have described a trade-off between hydraulic conductivity and wall mechanics [267,271,272]. As cells modify their walls to prevent water loss, they often reduce hydraulic permeability and wall extensibility. This creates a feedback loop: stiffening the wall protects against collapse and water loss but restricts the capacity for turgor-driven expansion [22,273,274]. The plant must strictly separate these processes within its zones: maintaining high *ϕ* in meristematic and elongation zones (apex), while rapidly decreasing *ϕ* and increasing Y in maturation zones to prevent water loss and revise its energy expenditure strategy [266]. Importantly, under drought, roots cell expansion is anisotropic (faster in one of three directions) because roots must elongate longitudinally to mine the soil in the search for moisture, while minimizing radial expansion [266,275]. This directional control is determined by the orientation of cellulose microfibrils, and the selective loosening of specific wall faces, therefore the drought response involves the specific modulation of cross-linking agents (like hemicelluloses and pectins) to favor cell elongation rather than widening [276,277].

### 6.2. Remodeling of CW Architecture

The physical state of the CW is determined by the collective action of numerous enzymes and proteins that modify its polysaccharide network [268]. Under drought, the expression and activity of these agents are altered to achieve either loosening (for growth) or stiffening (for protection) [265].

#### 6.2.1. Expansins

EXPs are non-enzymatic proteins that disrupt the non-covalent hydrogen bonds between cellulose microfibrils and matrix hemicelluloses (e.g., xyloglucans), allowing the polymers to slide relative to each other (a process termed “polymer creep”) [278]. This loosening is strictly pH-dependent, operating optimally in acidic conditions (typically optimal pH = 4–5) created by the plasma membrane proton pump, i.e., PM H^+^-ATPase [262]. Expansins are identified as positive regulators of drought tolerance, particularly in terms of root plasticity. Studies have shown that overexpression of expansin genes, namely *EXPA1* and *EXPA2*, enhanced drought tolerance, improved water retention, maintained cell turgor, and sustained root growth compared to wild-type plants [279,280,281] By increasing the wall extensibility, expansins allow cells to expand at lower turgor pressure, effectively compensating for the reduced water potential [282].

#### 6.2.2. Xyloglucan Endotransglucosylases/Hydrolases

While expansins facilitate physical sliding, XTHs remodel the covalent structure of the hemicellulose network [283]. Enzymes from the XTH family exhibit two distinct catalytic activities. Xyloglucan EndoHydrolase (XEH) activity irreversibly hydrolyzes xyloglucan, leading to CW weakening [284,285]. Xyloglucan EndoTransglucosylase (XET) cleaves the xyloglucan chain and transfers the cut end to another xyloglucan chain [286]. This is crucial for integrating the new wall material into the expanding network without losing mechanical integrity [283,287]. In drought-stressed roots, XET activity has been shown to loosen CWs in the elongation zone, facilitating continued growth [288,289]. The diversity of XTH encoding genes allows for highly specific spatial cells elongation regulation. Some isoforms are upregulated in roots to enable adaptive growth, while others may be downregulated in shoots to prevent wall creeping and reduce cell size [284,290]. Therefore, XTHs act as versatile remodeling agents that can support both stiffening and loosening of the CW.

#### 6.2.3. Pectins and Pectin Methylesterases

Pectins are CW heteropolysaccharides rich in galacturonic acids (Gal-A), forming the hydrogel matrix, in which cellulose and hemicellulose are embedded [291]. In plant cell walls, the most abundant pectin subtypes are homogalacturonans (HGs), which are crucial for establishing CW’s rheological properties [292]. In the apoplast, pectins, namely HGs, are demethylesterified by CW-localized PME enzymes [293]. Importantly, PME activity is regulated by Proteinaceous Pectin MEthylesterase Inhibitors (PMEIs), which bind to PMEs, forming a 1:1 complex [294,295]. Action of these enzymes can lead to two opposing outcomes: stiffening of CW by blockwise HG demethylesterification or its loosening by random HG demethylesterification [277]. When PMEs act on HGs in large contiguous blocks, the negatively charged carboxyl groups of HGs are exposed and can cross-link with Ca^2+^_,_ producing “egg-box” or “zipper” structures [293,296,297]. This creates a rigid pectate gel, which increases wall stiffness and reduces its permeability [298]. However, if demethylesterification is random, it can expose the HG backbone to degradation by PolyGalacturonases (PGs), leading to CW loosening [294]. It was shown that PME mode of action and its activity are pH-dependent [292,296]. At low apoplastic pH, PME activity rate decreases, resulting in low HG demethylesterification and reduction in the wall stiffening “egg-box”/“zipper” structures [299]. Under neutral/alkaline apoplast pH conditions, the activity of PMEs increases, causing higher content of block-wise demethylesterified HGs and CW stiffening [292]. Additionally, the stability of the PMEI-PME complex is enhanced in a more acidic environment, reducing PME activity [295,300]. The regulation of PMEs depends not only on pH, but also on multiple other factors, including Ca^2+^ level in the CW [297].

Researchers have found a link between the CW pectin demethylesterification rate and the “acidic growth” mechanisms in the regulation of elongation growth, adding another layer to the complexity of PME action [292,301]. The analysis of dark-grown hypocotyl cells revealed that PME-mediated pectin demethylesterification is involved in the control of anisotropic cell growth by promoting longitudinal wall elongation [302]. Under water stress, the CW plasticity was connected to the action of PMEs and low pectin content in the wall [303,304]. Additionally, drought stress often induces PME activity to mechanically reinforce cells against turgor loss [298,305]. In guard cells, the ABA-responsive *PME53* gene regulates the flexibility of the wall; its modulation is essential for the kinetics of stomatal closure [306,307]. As yet, the precise mechanism of regulation of the PME action on HGs under drought, and therefore its influence on CW plasticity, remains largely unexplored. However, there is sufficient evidence to hypothesize that PMEs may play a key role in modulating the CW rheology properties under water stress conditions to regulate cell elongation.

### 6.3. ABA and Auxin Interplay

The Acid Growth Theory remains the central paradigm explaining the initiation of CW loosening in the process of cell expansion [308]. This theory postulates that the extrusion of protons into the apoplast by the PM H^+^-ATPase lowers the extracellular pH (typically to ~4.5–5.5), which in turn activates specific wall-loosening proteins, such as expansins, and alters the structure of pectins [309,310,311]. Under drought stress, the regulation of PM H^+^-ATPase is a major control point for the differential growth of shoot and root tissues [312,313,314]. In shoots, drought response mechanisms, mediated by ABA signaling, generally suppress the PM H^+^-ATPase activity [315]. This suppression leads to apoplastic alkalinization (pH > 6.0), which inhibits expansin activity, promotes CW stiffening [316], and acts as a protective mechanism to arrest growth and close stomata [315]. In root tissues, the response is opposite. In the elongation zone of roots, proton pumping is often maintained or even enhanced to sustain the “acid growth” required for soil penetration [317]. The regulation of apoplastic pH involves a complex crosstalk between auxins and ABA [54,318]. According to the molecular basis of the Acid Growth Theory, auxins promote the activating phosphorylation of the C-terminal threonine residue of PM H^+^-ATPase [319]. During drought, however, ABA accumulation antagonizes this pathway, promoting the PM H^+^-ATPase deactivation through dephosphorylation [320]. Furthermore, ABA triggers the PM H^+^-ATPase internalization, affecting its transport to the plasma membrane via vesicle trafficking [321]. This antagonistic regulation allows for rapid switching between growth and its arrest [26,322,323].

In aerial tissues, the primary strategy is conservation of energy sources through the inhibition of cells expansion [14,324]. While this limits photosynthetic area, it significantly reduces the surface area available for transpiration and prevents wilting by mechanically reinforcing the cells [325]. Roots employ a distinct set of molecular mechanisms to continue elongation into dry soil [312]. Despite lower turgor, the extensibility of CW in the root apical zone increases significantly [266]. This is mediated by enhanced expression of expansins and XTHs that are not suppressed by ABA in this specific tissue [326,327]. The root tips exhibit a different sensitivity to ABA compared to the shoot. While high ABA inhibits growth, moderate hormone levels are actually necessary to maintain root elongation [317]. Roots actively curve toward environment with higher moisture (hydrotropism) by asymmetrical elongation of cortex cells in the elongation zone on the side of the root with lower water availability [328]. This process involves overcoming gravitropism by degradation of amyloplasts in the root cap, low ABA levels as well as the accumulation of SnRK2.2 and MIZU-KUSSEI 1 (MIZ1) proteins in the cortex cells [328,329]. While gravitropism is driven by auxin gradient, hydrotropism appears to function independently of auxin redistribution [330]. A 2023 study showed that auxins inhibit hydrotropism downstream of MIZ1 protein [331]. Furthermore, it has recently been proposed that, in the presence of ABA, MIZ1 mediates inhibition of polar auxin transport [75]. Overall, current evidence indicates that ABA and auxin interact antagonistically to regulate root orientation. While ABA signaling is required to suppress the polar auxin transport and initiate hydrotropism, auxins reciprocally function as a negative regulator that attenuates the hydrotropic response to prevent over-bending. The summary of ABA-mediated drought-tolerance strategies activated in shoots and in roots is featured in Table 4.

### 6.4. Methodological Frameworks in Plant Water Status

The evaluation of plant water status is not merely a matter of assessing its hydration; it is a complex analysis of thermodynamic, hydraulic, and metabolic adjustments occurring in plants to survive in a water deficient environment. Within the plant system, water is under pression, a metastable state maintained by the cohesive properties of water molecules and the structural integrity of xylem [332]. The quantification of this tension, defined as Ψ_w,_ serves as a foundational metric for the overall drought physiology [22]. Water potential constitutes the primary variable in plant water relations, integrating the effects of solute concentration, CW rheology properties, and hydrostatic pressure [23]. Accurate measurement of Ψ_w_ is essential to any discussion regarding drought stress severity, as it defines the driving force for water movement through the Soil–Plant-Atmosphere Continuum (SPAC) [23,332].

#### 6.4.1. Water Potential—Golden Standard Tools

One of the most well-known tools for water potential measurements within plant tissues is the Scholander pressure chamber [333]. The pressure chamber works by applying increasing positive air pressure to an excised leaf sealed inside the chamber until the sap is forced back to the cut surface of the protruding stem. The amount of external pressure required to balance the plant’s internal tension exactly equals the leaf’s negative water potential (Ψ_leaf/WL_) [334]. The pressure chamber is widely used both in the field and laboratory settings because it is portable and provides a direct and reliable measurement of xylem water potential, making it an accurate standard for both on-site assessments and controlled lab studies [333]. On the other hand, the pressure chamber is a destructive method, requiring a cutting of a leaf or stem from the plant for each measurement. Additionally, this process is manual and labor-intensive, which limits the number of samples that can be processed and prevents continuous, real-time monitoring of plant water status [335].

The thermocouple psychrometer is another well-established tool for studying plant water relations [336]. In this method, a tissue sample is sealed inside a small, thermally controlled chamber, allowing the air inside to reach vapor equilibrium with the sample. The sensor, typically a thermocouple consisting of two dissimilar conductors forming a junction, uses the Peltier effect to cool the junction and condense water vapor. It then measures the wet-bulb depression caused by the evaporation of water, providing a direct measure of the chamber’s humidity. This humidity measurement is directly related to the vapor pressure, which is used to calculate the water potential of plant tissue [337]. Psychrometers measure total water potential, accounting for both the physical tension in the xylem (pressure potential) and the effect of dissolved solutes (osmotic potential), thus offering a more complete physiological picture than the pressure chamber [338]. However, a major limitation of psychrometry is its extreme sensitivity to temperature fluctuations. Even minute thermal gradients (<0.001 °C) between the sample and the thermocouple can introduce substantial errors in the calculated water potential [336]. Importantly, in situ psychrometers can be clamped directly onto a live stem or leaves to measure water potential continuously without destroying the plant, while minimizing the temperature impact on achieved results [338].

#### 6.4.2. A New Era of Measuring Water Potential

Traditional methods for measuring water potential are reliable but labor-intensive and often provide only single “snapshots” in time. Modern tools focus on automation, continuous data collection, and non-destructive monitoring to capture the complete picture of plant water dynamics. Microtensiometer (e.g., FloraPulse) is a micro-electromechanical system (MEMS), which is embedded directly onto the trunk of woody plants [339]. Unlike psychrometers, which measure vapor, microtensiometers allow the xylem sap to equilibrate with a nanoporous membrane, measuring the tension electronically [340]. This direct monitoring of water status in real time over many months, with minimal maintenance, makes these devices ideal for agricultural research and crop management [341,342,343].

Hydrogel nanoreporters, also known as AquaDust, first described in 2021, represent cutting-edge technology for studying water relations in plant tissues. AquaDust consists of microscopic, dye-infused gel particles that infiltrate the interstitial spaces of living leaf tissue [344]. Once inside, these nanoparticles mechanically swell or shrink in equilibrium with the local water potential (Ψ_w_) of the surrounding plant cells. This physical change in volume alters the distance between the embedded dye molecules, causing a shift in their fluorescence emission spectrum via Förster Resonance Energy Transfer (FRET) [344]. Researchers can detect this optical signal using a fluorescent spectrometer to determine the precise water potential of the leaf without cutting or damaging it [345]. This technology enables non-destructive mapping of water stress gradients across a single leaf with high spatial resolution [346].

#### 6.4.3. Osmotic Potential and Turgor Dynamics

While total water potential defines the energy state of water within the plant, the physiological status of the cell is determined by its components: Ψ_s_ and Ψ_p_ [347]. Measurement of Ψ_s_ is most frequently performed using hygrometric techniques on tissue that has been frozen and thawed to rupture cell membranes, thereby eliminating turgor pressure [348]. Among these methods, vapor pressure osmometers (VPOs) and thermocouple psychrometers operate on the Peltier principle, measuring the wet-bulb depression of the sample [348,349]. VPOs offer high precision but often require mechanical extraction of sap onto filter paper, a step that introduces potential contamination artifacts [350]. In contrast, modern dew point potentiometers (e.g., WP4C) use a chilled-mirror sensor to detect the dew point [351]. The WP4C is advantageous for its rapid equilibration (typically < 40 min) and ability to measure whole leaf discs without the sap extraction, though it remains destructive, laboratory-bound instrument [352]. The second component of water potential—turgor pressure can be estimated using the aforementioned hygrometric tools as well using the subtraction method. This approach relies on the fundamental thermodynamic relationship, which is Ψ_p_ = Ψ_w_ − Ψ_s_ [353]. The protocol involves measuring the total water potential of fresh tissue, freezing the sample to induce lysis, and subsequently measuring the osmotic potential of the thawed tissue [354]. The difference between fresh and frozen tissues water potential values yields the turgor pressure.

Alternatively, osmotic potential and turgor dynamics can be derived simultaneously using a Pressure-Volume (P-V) curve, constructed using a pressure chamber and an analytical balance [351]. Unlike single-point hygrometric measurements, this method analyzes the relationship between water potential and tissue volume during a controlled dehydration sequence. By plotting the inverse of water potential (1/Ψ_w_) in MPa against the Relative Water Deficit (RWD) in % (calculated as 100-Relative Water Content (RWC)), it is possible to visualize the transition from turgor maintenance to turgor loss [355]. This plot reveals two distinct phases: a curved section, where turgor pressure is positive, and a straight linear section, which appears once the leaf has wilted (Ψ_p_ = 0) [356]. By mathematically extrapolating this “wilted line” back to the *y*-axis (which represents a fully hydrated leaf), researchers can define the osmotic potential at full turgor (π_o_) [351]. Consequently, the specific point at which the data deviates from this straight line and begins to curve marks the exact moment turgor pressure engages, defined as the turgor loss point (Ψ_TLP_) [352]. These parameters are critical for assessing drought tolerance, as more negative values of π_o_ and Ψ_TLP_ indicate the plant’s capacity to maintain cellular function under drier conditions [357]. Additionally, unlike hygrometric methods, P-V analysis allows the calculation of the bulk modulus of elasticity (ε) from the curved turgid phase of the plot, providing a metric of cell wall rigidity [351]. While the P-V curve is considered the gold standard due to its comprehensive dataset, it is significantly more labor-intensive than hygrometric methods, and generating a single curve often takes many hours, whereas tools like the WP4C can process multiple samples per day for osmotic potential alone [358].

## 7. Water Use Efficiency and Stomatal Regulation

Studies of drought tolerance and plant responses to dehydration often rely on the water use efficiency (WUE) index. Generally, WUE is defined as the ratio of carbon fixed by a plant to its water loss [359]. However, WUE is considered a highly complex concept as it is not a single, fixed value; its definition changes depending on the level at which it is measured [89]. Intrinsic WUE (WUE_i_) is measured in real-time at the leaf level as the ratio of CO_2_ assimilation to stomatal conductance *g*_s_ [360]. The WUE_i_ data is particularly valuable in the case of drought, as it provides a direct assessment of the physiological response to stress exerted by stomata over gas exchange [361]. The variation of WUE_i_, known as instantaneous WUE, also encompasses atmospheric water demand, which is primarily quantified as the vapor pressure demand (VPD) [359]. Instantaneous WUE shows the ratio of CO_2_ assimilation to transpiration; therefore, it directly quantifies how many molecules of CO_2_ are fixed by the plant (carbon gain) for each molecule of water it loses (water cost) through its stomata [362]. While both indexes use the rate of CO_2_ assimilation, WUEi separates the plant’s response to stress cues from atmospheric demand (i.e., VPD); however, the instantaneous WUE provides a broader environmental context which reflects the plant’s overall efficiency [359,363].

The long-term metric that shifts the focus from the instantaneous leaf physiology to the cumulative productivity of the plant under drought conditions can be calculated through the ratio of its total biomass accumulation to the cumulative transpiration, showing WUE at the whole-plant level (WUE_bio_) [89,364]. Moreover, WUE can be assessed in the scale of entire ecosystems (WUE_GPP_), which can range from a field or forest to global ecosystems [365]. Under mild drought conditions, WUE generally increases in plants, but this efficiency comes at the cost of a significant reduction in photosynthesis and overall growth, since the plant prioritizes water conservation over carbon acquisition [361]. WUE measured at the leaf scale provides valuable information about how plants regulate stomata conductance to respond and adapt to water shortage [366]. On the other hand, in the presence of water stress, the WUEbio assessment shows how effectively the plant has achieved a key compromise between maximizing growth and conserving water [367,368]. Importantly, the ecosystem-wide WUE_GPP_, showing the ratio of gross primary production (GPP) to water consumption, can be a very valuable tool for studying the effects of climate change on plants on the global scale. Cheng et al. [369] and Zhang et al. [69] hypothesize that PMEs may play a key role in modulating the CW rheology properties under water stress conditions to regulate the elongation growth.

### 7.1. Mechanisms Regulating Stomatal Aperture

Upon entering the guard cells, ABA initiates its canonical signaling pathway that acts as the main force closing the stomata [60]. Activation of SnRK2s triggers the efflux of anions, such as Cl^−^, NO_3_^−^, and malate (Mal^2−^), to the apoplast by activating anion channels located in the plasma membrane of guard cells, namely SLow Anion Channel 1 (SLAC1) and ALuminum-activated Malate Transporter 12 (ALMT12) [62]. Anion efflux causes plasma membrane depolarization, which activates voltage-gated Shaker-type outward-rectifying K^+^ channels (GORK), resulting in massive efflux of K^+^ [370]. The loss of key solutes leads to an increase in the intracellular water potential and outflow of water. Consequently, guard cells lose turgor and shrink, triggering stomata closure and transpiration restriction [60]. Moreover, SnRK2s directly contribute to ROS production and accumulation, which further promotes stomata closure [371,372].

**Figure 5:**
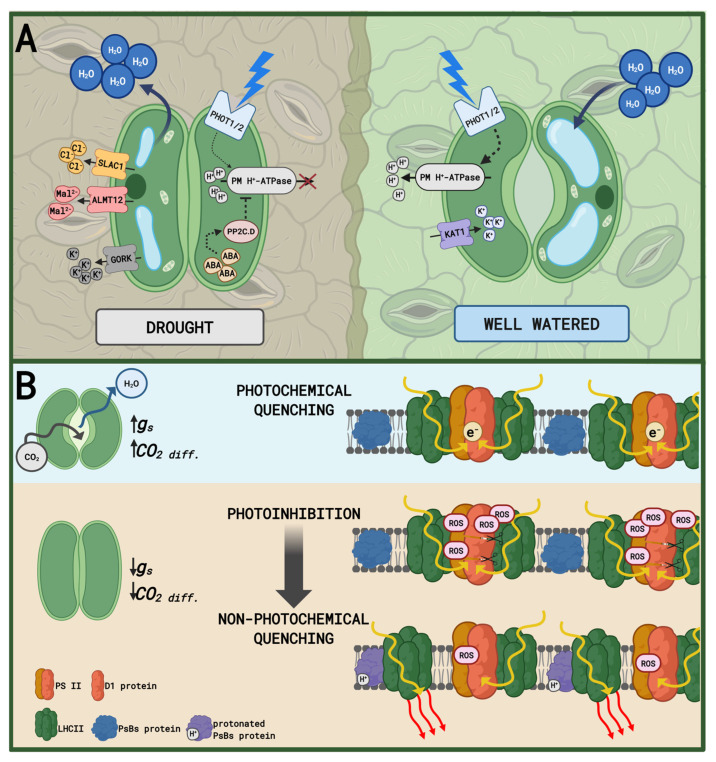
Mechanisms determining stomatal closure and photoprotective mechanisms activated during drought. (**A**) Drought-induced stomata closure with silmutanious exposure to light (left) and stomata opening stimulated by blue light under normal conditions (right); (**B**) The quenching effectivenes under normal conditions, which promote stomata opening (upper) and under drought, when stomata closure is stimulated (down). Created in BioRender. Michalak, A. (2025) https://BioRender.com/cmlm5kd. Accessed on 29 November 2025.

On the other hand, stomatal aperture is regulated by the action of PM H^+^-ATPase. Its activation leads to plasma membrane hyperpolarization, triggering massive ion influx, which increases turgor of guard cells and induces stomata opening [373]. However, in guard cells, stress factors like water deficiency led to the PM H^+^-ATPase deactivation, which is directly mediated by PP2Cs, a core ABA signaling protein phosphatases described above [57,374]. Ultimately, the coordinated effects of ion efflux, reduced proton pump activity, and ROS accumulation lead to a loss of guard cell turgor and, consequently, to stomatal closure [60]. While the ABA pathway serves as the central chemical signaling for stomatal closure during drought and osmotic stress, stomatal aperture size is the effect of integrating multiple antagonistic and synergistic signaling pathways [375,376,377]. Blue light (400–500 nm) promotes stomatal opening via photoreceptors—PHOTotropin 1 and 2 (PHOT1/2), which activate PM H^+^-ATPase through phosphorylation [378,379]. This activation leads to plasma membrane hyperpolarization and the subsequent influx of K^+^ mediated by voltage-gated Shaker-type inward-rectifying K^+^ channels, such as KAT1, increasing guard cells turgor [370,380]. The ABA pathway, however, dominates during water stress, directly counteracting this light-activated signaling [381]. The protein phosphatases PP2C.D, involved in ABA signaling, directly inhibit the blue light-induced activation of PM H^+^-ATPase (Figure 5A) [57,374]. This inhibition ensures that the plant prioritizes water conservation over carbon gain, even in bright sunlight. The relationship between WUE and ABA is key for plant survival strategies under drought conditions, since ABA acts as the primary hormonal signal that fine-tunes stomatal aperture to maximize WUE by balancing CO_2_ influx and water loss. Studies have shown that both high endogenous ABA level and exogenous ABA application increased plant WUE and led to sustained biomass maintenance [381,382,383,384].

### 7.2. Photosynthetic Limitations Caused by Closed Stomata

The initial ABA-mediated stomatal closure, as a part of the plant’s survival tactic, increases WUE by prioritizing water conservation. However, this high WUE state comes at the cost of a reduced CO_2_ diffusion rate from the atmosphere to the leaves, which impairs the process of photosynthesis [385,386]. CO_2_ shortage not only deprives Rubisco, a key photosynthesis enzyme, of its substrate, but also induces photorespiration, which reduces carbon fixation efficiency and overall photosynthetic productivity [386,387]. Moreover, water stress has been linked to a decrease in ATP synthase abundance, leading to reduced synthesis of ATP, which provides the energy necessary for carbon fixation during the Calvin cycle and Rubisco activation [388,389]. Additionally, under drought conditions, the stomata closure causes photoinhibition, as the light energy absorbed by photosystems exceeds the capacity of carbon fixation [390,391]. This excess energy generates ROS, which damage key cellular structures, such as D1 protein in PSII, contributing to further decrease in photosynthesis efficiency [391,392]. However, plants can activate photoprotective mechanisms, including Non-Photochemical Quenching (NPQ), to dissipate the excess of light energy [391,393]. Under water stress, prevention of PSII damaging during NPQ involves Light-Harvesting Complex II (LHCII) migration from PSII and its subsequent conformational change induced by the protonated PsbS (Photosystem II subunit S) protein and zeaxanthin [394,395]. This change causes a switch of LHCII from its highly efficient light-capturing state to a “quenching” state, in which excess energy from chlorophyll excitation is released as heat rather than being transferred to the PSII reaction center, thus preventing the formation of harmful ROS (Figure 5B) [393,396,397]. Importantly, during acclimation to water stress, plants undergo osmotic adjustment, which allows for partial opening of the stomata, thereby mitigating carbon shortage [398,399]. This adjustment involves stimulation of synthesis and accumulation of various osmoprotectants or compatible solutes, causing the Ψ_w_ of cells to decrease [400,401].

### 7.3. Evaluation of the Stress-Imposed Damage to the Photosynthetic Apparatus

In plant cells, the photosynthetic apparatus is one of the sites susceptible to drought-related damage due to the disruption of linear electron flow and the resulting accumulation of excess excitation energy [389]. Assessing a plant’s photosynthetic capacity is a key source of quantitative information about the processes initiated to manage this energy imbalance under stress [402]. By combining the analysis of chlorophyll fluorescence to monitor PSII efficiency with the biochemical profiling of leaf pigments, it is possible to better understand plant physiology during drought response [403].

#### 7.3.1. Assessment of PSII Efficiency and Photoprotection Using PAM Fluorometry

Chlorophyll *a* fluorescence (ChlF) analysis is one of the most widely used techniques in drought research due to its non-invasive nature and rapidity [404]. The standard assessment of PSII status by measuring ChlF often involves a Pulse-Amplitude-Modulation (PAM), which is a well-established method for evaluating plant photosynthetic performance under water deficit [405]. Its primary application in drought research lies in its ability to detect physiological stress before visible symptoms, such as wilting or chlorosis, appear [406]. Moreover, PAM fluorimetry distinguishes between the energy used for photochemical reactions and the excess energy dissipated as heat [407].

To provide a comprehensive physiological profile of plants under drought, several key parameters are commonly assessed in contemporary studies including maximum quantum yield of PSII (Fv/Fm), photochemical quenching (qP), effective quantum yield of PSII (ΦPSII), redox state of the plastoquinone pool (qL), and NPQ [408]. Fv/Fm, measured in dark-adapted leaves, represents the maximum quantum efficiency of PSII and is a critical indicator, as its value typically decreases under drought, reflecting the occurrence of photoinhibition induced by water stress [409,410]. This parameter is also useful for evaluating both the drought resistance of plants and their recovery ability [411]. The qP coefficient estimates the proportion of open PSII reaction centers and indicates a disruption of linear electron flow. Under water-deficient conditions, a decrease in this value was observed in stressed plants [412,413]. ΦPSII, measured under illuminated conditions, assesses the actual operating efficiency of electron transport in the light, and its decline under drought is attributed to the inactivation of PSII reaction centers activated as a photoprotective response [409,414]. The qL coefficient provides insight into the redox state and the contribution of open PSII reaction centers, with lower values observed in drought-stressed plants [414,415,416,417]. NPQ is a crucial photoprotective parameter measured in drought studies, and while its value often increases in response to drought, it is important to note that under stress conditions, NPQ is highly dependent on the light intensity during the experiment [393,418].

#### 7.3.2. Quantification of Photosynthetic Pigments and Anthocyanins

Quantitative assessment of leaf pigments, such as chlorophylls (*a* and *b*), carotenoids, and anthocyanins, remains a fundamental assay in drought research [8,419]. Beyond measuring the plant’s capacity for light harvesting, pigment profiling serves as a sensitive biochemical marker for oxidative stress and leaf senescence [420,421]. Under water deficit, the equilibrium between pigment synthesis and degradation is disrupted [422,423]. Monitoring these changes allows researchers to distinguish between regulated acclimation (e.g., reducing antenna size to prevent overexcitation) and irreversible senescence (photooxidative destruction of thylakoid membranes) [424,425].

Chlorophylls and total carotenoids are typically extracted in organic solvents, and their concentration can be determined spectrophotometrically [426]. Because photosynthetic pigments have distinct absorption maxima, their concentrations can be determined simultaneously from a single extract using appropriate equations. The choice of solvent for pigment extraction is important, as its polarity can shift the peak absorbance of each pigment, which requires the use of specific equations to obtain accurate calculations [427]. For example, using the standard equations for 80% acetone, absorbance is measured at 663 nm for (Chl *a* peak), 647 nm (Chl *b* peak), and 470 nm (total carotenoids peak). Because the absorption spectra of Chl *a* and Chl *b* overlaps significantly, simultaneous equations are used to mathematically separate their contributions [427]. Total carotenoids are then calculated by measuring absorbance at 470 nm and subtracting the specific interference contributions of the calculated chlorophylls [426]. Anthocyanins, being water-soluble vacuolar pigments, require a separate extraction protocol, typically using acidified alcohol (e.g., methanol/ethanol with HCl) to maintain stability and spectral integrity [428,429,430]. Their quantification relies on absorbance measured near the peak of 530 nm (A_530_) [431]. In drought-stressed leaves, whose tissues may be tough, and extraction can be challenging, it is important to apply a correction factor to ensure accurate quantification. This is typically done by subtracting the absorbance at 657 nm (A_657_) to account for degradation products or overlapping chlorophylls that may have entered the acidic extract [431].

Drought stress typically leads to a decline in total chlorophyll content due to the upregulation of chlorophyllase activity and ROS-mediated destruction of pigments [417,423,432]. Unlike chlorophyll *a*, chlorophyll *b* is found exclusively in light-harvesting antenna complexes. Therefore, under drought conditions, a decrease in chlorophyll *b* content may indicate that plants reduce their light-harvesting capacity by diminishing the number of these complexes to prevent oxidative stress-related damage [433,434]. As mentioned before, under water stress carotenoids are one of the essential photoprotective agents that scavenge ROS and dissipate excess energy [435], and their content is affected by multiple factors, including species, age, genotype, duration and intensity of stress, and the light intensity [436,437]. Consequently, while some studies report carotenoid accumulation as a response to water deficit, others show a decrease in carotenoid content [438,439,440,441]. However, increased carotenoid level may be associated with drought resilience, reflecting the activation of damage-mitigating machinery to combat oxidative stress [12,442,443]. In drought-tolerant genotypes, the carotenoid/chlorophyll ratio may increase, which could indicate a shift toward photoprotection rather than light collection, potentially helping to preserve membrane integrity during dehydration [444,445]. Under drought conditions, anthocyanin accumulation also helps protect chloroplasts from excessive light and UV radiation. Additionally, these pigments act as powerful antioxidants to scavenge ROS [446]. Upregulation of anthocyanin synthesis has been correlated with enhanced drought tolerance in some plant species [447,448,449]. For example, the purple stem genotype *Brassica napus* plants with a purple stem genotype, which exhibit a 50-fold increase in anthocyanin pigmentation, demonstrate greater structural and functional integrity of mesophyll cells, higher photosynthetic efficiency, and improved ability to mitigate oxidative stress under drought conditions compared to the green stem *B. napus* genotype [450].

## 8. Integrating Multi-Omics and High-Throughput Phenotyping in Drought Research

For many years, the understanding of plant responses to drought stress relied primarily on traditional physiological and molecular techniques. However, fully capturing the complexity of stress adaptation has necessitated a development of broader, more precise, and system-level approaches. Consequently, for the past few decades, drought research has increasingly focused on the integration of well-established methods with high-throughput omics analyses [451]. Firstly, omics technologies were mainly used independently, with researchers adding either genomics (analysis of genomes), transcriptomics (gene expression profiling), proteomics (identification and quantification of proteins), or metabolomics (profiling of metabolites/signaling molecules) analysis to their studies [451,452]. Unfortunately, a single-omics approach is not sufficient to fully comprehend the multilayered and highly complex plant response to stress. Therefore, the future of drought research currently lies with integrative omics. This approach, also known as multi-omics, combines the above-mentioned high-throughput methods with phenomics (phenotyping), interactomics (interaction studies), and epigenomics (gene regulation and various adaptations) [452,453,454]. This allows research to shift from simple observations of certain traits and mechanisms to a comprehensive understanding of plant behavior under water deficit conditions.

Integrative omics are especially useful in developing drought-tolerant plants. Many studies used multi-omics to analyze various crop species including, rice [451], grain legumes [455], tea [456], sweet potato [457], tomato [458], wheat [459], or corn [460] to identify either plant varieties with lower water requirements or crucial molecular pathways that will enable the creation of drought-resistant plants.

To fully unlock the potential of multi-omics, a technique which generates large amounts of data, research must simultaneously focus on the development of support systems based on bioinformatics and artificial intelligence (AI). Usually, once the data is generated from any omics technique and initially processed, it needs to be integrated, functionally analyzed, and networked, which requires both Machine Learning algorithms and computational biology [452]. Importantly, studies involving integrative omics can move beyond simple data gathering and correlation, towards identifying key genes, proteins, metabolites, or other regulators that cooperate and participate in the drought stress response [452,461]. This approach, hopefully in the near future, will allow, firstly, to improve phenotypic predictions and then to construct predictive models for targeted plant breeding and engineering [462].

The current bottleneck in drought research is no longer genotyping, but phenotyping. High-throughput phenotyping (HTP) has emerged as a critical complement to omics, enabling the efficient, non-invasive collection of plant traits across large populations to address the limitations of traditional phenotypic methods [463,464]. Advancements in sensor technology allow us to assess drought tolerance directly in the field conditions, using various imaging systems [465]. RGB indices, for instance, correlate strongly with grain yield and leaf yellowing, providing reliable stress assessments even in dense canopies [466,467,468]. Similarly, Near-Infrared (NIR) spectroscopy serves as a cost-effective tool for detecting early signs of drought stress and identifying tolerant genotypes in crops like wheat and grapevine [469,470,471]. Beyond visual assessments, thermal imaging detects temperature increases resulting from decreased stomatal conductance, allowing for the real-time observation of plant water status [472,473,474]. Complementing this, spectral imaging captures electromagnetic radiation to track physiological changes, such as chlorophyll content, which varies significantly with soil moisture levels [465,475]. For structural analysis, LiDAR technology generates high-resolution 3D models to precisely measure canopy architecture, plant height, and biomass under water stress [476,477]. The integration of these imaging technologies with unmanned aerial vehicles (UAVs), mobile robots, and AI has revolutionized data collection, enabling autonomous monitoring and improved trait quantification [473,478,479]. Whether applied in large-scale research infrastructures or commercial precision agriculture, HTP facilitates early intervention and accelerates the development of drought-resilient cultivars [480,481]. Ultimately, HTP and multi-omics approach bridge the gap between phenotypic data and actionable agricultural strategies, providing robust gene-to-phenotype regulatory networks [482].

## 9. Conclusions and Future Perspectives

Given the intricacies of plants’ drought responses, a comprehensive analysis of plant physiology, integrating diverse methodological approaches, is inevitable to fully decipher survival mechanisms. While traditional breeding of plants showing drought-resilient traits remains fundamental, it is often too slow to keep pace with rapid environmental changes. Therefore, the most effective immediate strategy seems to be molecular screening to identify currently existing resilient plants’ varieties and incorporate them into agriculture. However, looking further ahead, the accelerating rate of global temperature rise suggests that this may not be sufficient; consequently, genetic engineering of new plant varieties will possibly become an inevitable approach to ensure long-term crop resilience. Nevertheless, more research is still required to successfully generate such drought-resilient genetically modified crops [483]. For this reason, to achieve food safety and preserve biodiversity for the future, the studies of plants’ drought response should take into consideration following aspects:

- Accurately quantifying and standardizing drought severity on plants (e.g., by measuring soil water content or Ψ_w_) to reflect realistic deficits, thereby ensuring translational validity of laboratory results. An alternative could be to use a liquid medium containing PolyEthylene Glycol (PEG); however, this method may not fully reflect the water stress that plants experience in nature.
- To enable valid comparisons across independent studies, the research framework should include a minimum set of crucial physiological parameters, such as leaf water potential, photosynthetic efficiency, oxidative stress parameters, and pigment analysis. This standardized baseline is essential for distinguishing the actual physiological status of a stressed plant.
- Categorizing the studied species or cultivars within established drought resistance strategies would enable the translation of research data into practical, useful information for other scientists and breeders.
- Functional redundancy within transcription factor families (e.g., WRKY, NAC, DREB) often masks the impact of single-gene modifications. Future research must incorporate multi-omics approach to map regulatory hubs, enabling simultaneous editing of multi-genes or trait stacking. Manipulating entire gene clusters is necessary to bypass redundancy and engineer robust drought resilience.
- In nature, drought stress rarely occurs in isolation. Therefore, experimental designs should consider realistic combinations of various factors, such as water scarcity, high irradiance, heat waves, and elevated atmospheric CO_2_ levels, projected over the coming decades, providing a comprehensive picture of the climatic relationships relevant to plant survival in future agroecosystems.

## Figures and Tables

**Table 1:** Crosstalk between ABA and other phytohormones under drought stress.

Phytohormone	Interaction Type	Mechanism of Action	References
Cytokinins (CKs)	Antagonistic	Stomatal aperture: CKs naturally promote stomatal opening. Under drought, CK levels decrease, resulting in downregulation of opening signals and sensitization of guard cells to ABA-induced closure. Growth balance: a low CK/high ABA ratio inhibits shoot growth while maintaining root activity, optimizing the root-to-shoot ratio for survival.	[73]
Auxins	Complex	Deep Rooting: ABA promotes local auxin synthesis in root tips to steepen growth angles (gravitropism), enabling reaching deep water. Hydrotropism: To orient towards moist areas, ABA modifies auxin transport, temporarily suppressing gravitropism. Stomata aperture: Auxins generally promote opening. ABA must suppress the sensitivity of guard cells to auxin to ensure timely closure.	[26,74,75]
Brassinosteroids (BRs)	Antagonistic (mostly)	BRs promote cell elongation and stomatal opening. High ABA levels are required to override this signal. Note: Exogenous BRs application can improve resistance by stimulating ABA biosynthesis.	[76,77,78,79]
Jasmonic Acid (JA)	Synergistic	JA works together with ABA to stimulate stomatal closure. Additionally, JA stimulates de novo ABA biosynthesis.	[80,81,82,83]
Strigolactones (SLs)	Synergistic	SLs increase the sensitivity of stomata to ABA. SL-deficient mutants often fail to close stomata efficiently even when ABA is present.	[84,85]
Melatonin	Antagonistic/Modulatory	By scavenging ROS and alleviating oxidative stress, it downregulates ABA biosynthesis gene *NCED3* and promotes ABA catabolism, preventing excessive senescence and allowing growth recovery.	[86,87]

**Table 2:** Representative TFs and their function in Arabidopsis, rice, maize, wheat, tomato, and grapevine.

Family of TFs	Plant Species	Protein Name	Regulation	Function/Plant Characteristics	References
bZIP	*Arabidopsis thaliana*	AtAREB1/AREB2/ ABF3/ABF1	Positive	Master transcription factor in ABA-dependent signaling	[96]
	*Oryza sativa*	OsABF1	Positive	Activator in ABA-dependent gene expression	[97]
		OsABF2/bZIP46	Positive	Activator in ABA-dependent gene expression, regulation of WRKY TFs	[97]
		OsbZIP23/62	Positive	Activator in ABA-dependent gene expression	[97]
		OsbZIP72	Positive	Activator in ABA-dependent gene expression, including LEA	[98]
	*Zea mays*	ZmABP9	Positive	Activator in ABA-dependent gene expression including *KIN1*, *COR15A*, *PP2C*, *AZF2*, ROS regulation	[99]
		ZmbZIP72	Positive	Activator in ABA-dependent gene expression including *RAB18*, *RD29B*, *HIS1-3*	[99]
		ZmABI5	Negative	Regulation of the *CAT1*, *APX* and *NtERD10A*, *B*, *C*, *D* gene expression	[99]
	*Triticum aestivum*	TaABP1	Positive	Activator in ABA-dependent gene expression	[97]
		TaABL1	Positive	Activator in ABA-dependent gene expression, regulation of stomatal movement, accumulation of osmolytes	[97]
		TaAREB3	Positive	Activator in ABA-dependent gene expression	[97]
		Wabi5	Positive	Activator in ABA-dependent gene expression, e.g., LEA	[97]
	*Solanum lycopersicum*	SlAREB1	Positive	Activator in ABA-dependent gene expression including *AtRD29A*, *AtCOR47*, and *SlCI7-like dehydrin*	[100]
		SlbZIP1	Positive	Activator in ABA-dependent gene expression, regulation of chlorophyll content and CAT activity	[101]
	*Vitis vinifera*	VlbZIP30	Positive	Activator in ABA-dependent gene expression including stress marker genes and ABA core signaling components	[102]
		VlbZIP36	Positive	Activator in ABA-dependent gene expression, increased activity of antioxidant enzymes	[103]
		VvABF2	Positive	Activator in ABA-dependent gene expression including *RAB18*, *LEA*, *RD29B* modulation of antioxidative enzymes activity	[104]
MYB	*Arabidopsis thaliana*	AtMYB60	Negative	Stomatal movement	[105]
		AtMYB96	Positive	Stomatal movement, modulation of auxin homeostasis during lateral root development, wax synthesis	[105,106]
		AtMYB77	Positive	Auxin signaling-dependent lateral root growth	[105]
		AtMYB2	Positive	Activator in ABA-dependent gene expression, including *AtRD22* and *AtADH10*	[105,106]
		AtMYB15	Positive	Expression enhancment of genes involved in drought-response (*AtADH1*, *RD22*, *RD29B*, *AtEM6*), ABA biosynthesis (*AtABA1*, *AtABA2*) and signaling (*AtABI3*)	[107]
		AtMYB44	Positive	Auxin signaling-dependent lateral root growth, cross-talk between SA and JA (direct regulation of *AtWRKY70)*	[105,106]
		AtMYB20	Negative	Stomatal movement	[105]
	*Oryza sativa*	OsMYB48-1	Positive	Proline biosynthesis, ABA accumulation, expression enhancement of genes involved in ABA biosynthesis (*OsNCED4*, *OsNCED5*), signaling (*OsPP2C68*, *OsRK1*) and response (*OsRAB21*, *OsLEA3*, *OsRAB16C* and *OsRAB16D*)	[105]
		OsMYB60	Positive	Wax synthesis	[106]
		OsMYB1-R1	Negative	Proline biosynthesis	[108]
		OsMYB2	Positive	ABA signaling, stomatal movement, regulation of gene expression of *OsLEA3*, *OsRAB16A*, and *OsDREB2A*	[105,108]
	*Zea mays*	ZmMYB30	Positive	Upregulation of stress-responsive genes *ABF3*, *ATGolS2, AB15, DREB2A, RD20, RD29B, RD29A*, and *MYB2*	[99]
		ZmMYB56	Positive	Stomatal movement regulation via transcriptional regulation of *ZmTOM7*	[109]
	*Triticum aestivum*	TaMYB3-R1	Positive	Stomatal movement	[105]
		TaMYB2	Positive	Upregulation of stress-related genes *AtDREB2A*, *AtRD29B*, *AtRD22*, *AtCOR15*, *AtRab18* and *AtABI2*	[105]
		TaMYB19	Positive	Accumulation of osmolytes, upregulation of stress-related genes including *AtRD29A*, *AtRD22* and *AtMYB2*	[105]
		TaMYB30-B	Positive	Accumulation of osmolytes, upregulation of stress-responsive genes including *AtRD29A* and *AtERD1*	[105]
		TaMYB33	Positive	Maintenance of osmotic balance, increased ROS scavenging	[110]
		TaODORANT1	Positive	Upregulation of the expression of ROS- and stress-related genes	[111]
		TaMYB44-5A	Negative	Downregulation of the *TaRD22-3A*, drought- and ABA-responsive gene expression	[112]
		TaPIMP1	Positive	Increased drought-tolerance and SOD activity, upregulation of defense- and stress-related genes, including *RD22*	[105]
	*Solanum lycopersicum*	SlMYB49	Positive	Reduced ROS accumulation	[113]
		SlMYB1L	Positive	Stomatal movement, proline and H_2_O_2_ homeostasis	[114]
		SlMYB78-like	Positive	Regulation of chlorophyll biosynthesis, photosynthesis, ABA biosynthesis and response genes	[115]
	*Vitis vinifera*	VvMYB60	Negative	Stomatal movement	[105]
		VyMYB24	Positive	Regulation of root development, antioxidant enzymes, proline accumulation	[116]
		VvMYB14	Positive	Regulation of POD and CAT expression	[117]
AP2/ERF (DREB)	*Arabidopsis thaliana*	AtDREB1A	Positive	Regulation *AtRD29A* and *AtCOR15A* expression	[118]
		AtDREB2A	Positive	Core regulator of ABA-independent drought genes	[119]
		AtTINY	Positive	Stomatal movement, alleviation of BES1 repression of drought-responsive genes.	[120]
	*Oryza sativa*	OsDREB1A	Positive	Accumulation of proline, maintenance of chlorophyll, regulation of RWC and ion leakage	[121]
		OsDREB2A	Positive	Core regulator of ABA-independent drought genes	[119]
	*Zea mays*	ZmDREB1A	Positive	Expression of drought-responsive genes in both the ABA-independent and ABA-dependent pathways	[99]
		ZmDREB2A	Positive	Key regulator of the dehydration-responsive regulon	[99]
		ZmDBF3	Positive	Improvement of drought tolerance	[99]
		ZmDBP3/4	Positive	Improvement of drought tolerance	[99]
	*Triticum aestivum*	TaDREB1	Positive	Improvement of drought tolerance	[122]
		TaAIDFa	Positive	Improvement of drought tolerance	[122]
		TaDREB2	Positive	Regulation of IAA	[122]
	*Solanum lycopersicum*	SlDREB1	Positive	Accumulation of soluble sugars and osmolytes Increased response to drought-stress	[123]
		SlDREB2	Positive	Regulation of stress signaling pathways and proline synthesis	[123]
		SlDREB3	Negative	Alteration of ABA signaling by negative regulation of the ABA pathway	[123]
NAC	*Arabidopsis thaliana*	AtANAC096	Positive	Interaction with AtABF2 and AtABF4 in the ABA signaling pathway, regulation of *AtRD29A* gene expression	[124]
		AtANAC019/055/072	Positive	Improvement of drought tolerance	[125]
		AtRD26	Positive	Activator in ABA-dependent gene expression, inhibitor of BES1 expression	[126]
		AtATAF1	Positive	Regulation of ABA biosynthesis through AtNCED3 and SnRK1	[127]
	*Oryza sativa*	SNAC1	Positive	Stomatal movement, improvement of drought tolerance by regulation of *OsPP18* (PP2C) and ROS scavenging enzymes expression in ABA independent pathway	[124,128]
		OsNAC5	Positive	Transcriptional activation of stress-responsive genes, lignin biosynthesis, improvement of lateral root formation	[124]
		OsNAC6	Positive	Improvement of lateral root formation, upregulation of the expression of genes involved in membrane modification, NA biosynthesis, and GSH relocation.	[124,129]
		OsNAC78	Positive	Maintenance of ROS homeostasis through activation of *OsGSTU37*	[124]
	*Zea mays*	ZmSNAC1	Positive	Enhancement of tolerance to drought stress at the germination phase	[99]
		ZmNAC55	Positive	Enhanced tolerance to drought stress through upregulation of genes including *RD29B*, *LEA14*, *RD17*	[99]
		ZmNAC111	Positive	Upregulation of drought stress–responsive gene expression	[130]
		ZmNAC20	Positive	Stomatal movement, activation of drought-responsive genes	[131]
		ZmNAC45/72/18/51	Positive	Improvement of drought tolerance	[99]
	*Triticum aestivum*	TaNAC6-3B	Positive	Regulation of NCED, ABA and drought-responsive genes, including LEA	[132]
		TaNAC69-5A	Positive	Improvement of root architecture and drought tolerance	[122]
		TaNAC2	Positive	Stomatal movement, improvement of root architecture and drought tolerance	[122]
		TaNAC29	Positive	Improvement of drought tolerance by reduction in ROS accumulation	[122]
		TaNAC8-6A	Positive	Stomatal movement	[122]
	*Solanum lycopersicum*	SlJUB1	Positive	Regulation of *SlDREB1* and *SlDREB2* expression and proline synthesis	[133]
		SlNAC4	Positive	Improvement of drought tolerance	[133]
		SlNAC6	Positive	Accumulation of proline and antioxidant enzymes	[134]
		SlNAP1	Positive	Regulation of GA and ABA synthesis	[135]
		SlSRN1	Negative	Gene silencing increases drought tolerance	[136]
	*Vitis vinifera*	VvNAC17	Positive	Accumulation of antioxidative enzymes and anthocyanin, regulation of drought-related gene expression including *VvDREB1A*, *VvDREB2A*, *VvRD29A*	[137]
		VvNAC33	Positive	Regulation of the antioxidant enzymes expression including *VvCAT1*, *VvCu/ZnSOD*, and *VvPOD4*	[138]
WRKY	*Arabidopsis thaliana*	AtWRKY57	Positive	Upregulation of *AtRD29A*, *AtNCED3*, and *AtABA3* expression	[139]
		AtWRKY53	Negative	Stomatal movement: inhibition of stomatal closure via reduced H_2_O_2_ content, facilitation of stomatal opening by starch degradation	[140]
		AtWRKY63 (ABO3)	Positive	Regulation of RD29A and COR47A in ABA signaling	[141]
		AtWRKY40	Negative	Expression inhibition of multiple ABA-induced genes including AtABF4, *AtABI4*, *AtABI5*, *AtDREB1A*, *AtMYB2*, and *AtRAB18*	[142]
		AtWRKY18	Negative	Suppression enhancement of *AtABI4* and *AtABI5* transcription induced by WRKY40	[142]
	*Oryza sativa*	OsWRKY11	Positive	Activator of the drought-responsive gene transcription, e.g., *OsRAB21*	[143]
		OsWRKY5	Negative	Suppresion of OsMYB2 expression with downregulation of its downstream genes (*OsLEA3*, *OsRAB16A,* and *OsDREB2A*)	[108]
		OsWRKY47	Positive	Activation of genes involved in inhibition of stress-induced senescence	[144]
		OsWRKY55	Negative	Negative modulation of drought response via joined transcriptional cascade with OsAP2-39	[145]
	*Zea mays*	ZmWRKY58	Positive	Protection of cell membrane integrity, participation in the ABA and Ca^2+^ signaling pathway	[146]
		ZmWRKY40/106	Positive	Activation of *DREB2B*, and *RD29A* expression in transgenic plants	[146]
	*Triticum aestivum*	TaWRKY33	Positive	Improvement of drought tolerance in transgenic plants via ABA synthesis and transduction pathways.	[147]
		TaWRKY75-A	Positive	Upregulation of JA biosynthetic genes *AtLOX3* and *AtAOC1*	[148]
		TaWRKY44	Positive	Indirect activation of genes associated with cellular antioxidant systems and stress response	[149]
		TaWRKY2-1D	Positive	Regulation of the expression of *TaPOD*, *TaCAT*, *TaSOD*(*Fe*), and stress-related *TaP5CS*	[150]
		TaWRKY133	Negative	Downregulation of transcription of drought-responsive (*DREB2A*, *RD29A*, *RD29B*, *ABF1*, *ABA2*, *ABI1)* and antioxidant enzymes: *SOD(Cu/Zn)*, *POD1*, and *CAT1* genes	[151]
	*Solanum lycopersicum*	SlWRKY75	Positive	Enhancement of drought tolerance via JA signaling and regulation of *SlARF5* and *SlTRY* expression responsible for lateral roots and trichome formation	[152,153]
		SlWRKY8	Positive	Accumulation of osmotic substances, upregulation of stress-responsive genes *SlAREB*, *SlDREB2A,* and *SlRD29*.	[154]
		SlWRKY17	Positive	Upregulation of ROS detoxification-related and drought-responsive genes	[155]
		SlWRKY81	Negative	Regulation of H_2_O_2_–mediated stomatal movement	[156]
	*Vitis vinifera*	VvWRKY48	Positive	Increased activity of the antioxidant enzymes, upregulation of the expression of stress-related genes	[157]
		VvWRKY18	Negative	Increased ROS accumulation, lowered activity of antioxidant enzymes, increase in stomatal density	[158]
		VvWRKY13	Negative	Reduced content of osmolytes, increased ROS accumulation, reduced drought-related gene expression	[159]

**Table 3:** Methods used for studying ROS-related changes in plants.

Type of Detection	Name of the Method	Measured Parameters	Characteristics	Localization	References
Indirect methods (in vitro)					
Spectrophotometry	Biochemical estimation	chlorophylls, anthocyanins, MDA, proline	Simple, fast, cost-effective, low sensitivity	Not applicable	[236,239]
	Enzymatic assays	SOD, CAT, APX, GR, MDHAR, DHAR		Not applicable	
	Non-enzymatic antioxidant assays	AsA, GSH, vitamins, flavonoids		Not applicable	
Chromatography	HPLC	Proline, glycine betaine, sugars	highly specific, sensitive, time-consuming sample preparation, requirement of complex instrumentation	Not applicable	[236,239]
2. Indirect methods (in vitro and in vivo)					
Non-fluorescent/colorimetric probes	DAB	H_2_O_2_	Simple, low specificity, irreversible	Nontargeted	[219,236,238]
	NBT	O_2_^•−^		Nontargeted	
Fluorescent probes	CM H_2_DCF-DA	ROS in general	Irreversible, low specificity, possibility of photooxidation	Intracellular	
	OxyBurst Green	ROS in general	Irreversible, low specificity, possibility of photooxidation	Extracellular	
	DHR	ROS in general	Irreversible, low specificity	Intracellular	
	BES H_2_O_2_-Ac	H_2_O_2_	Irreversible, slow reactivity	Intracellular	
	Amplex Red	H_2_O_2_	Irreversible, pH sensitive, possibility of photooxidation	Extracellular	
	boronate-based	H_2_O_2_	Irreversible, high sensitivity, high stability, may react with other ROS	Intracellular, Extracellular	
	NBCD	H_2_O_2_	Irreversible, high specificity, high stability	Intracellular	
	DHE	O_2_^•−^	Irreversible, may react with other ROS	Intracellular	
	MitoSOX	O_2_^•−^	Irreversible, may react with other ROS	Mitochondria	
	DanePy	^1^O_2_	Irreversible, may react with other ROS, possibility of photobleaching, high photosensitivity	Intracellular, Chloroplasts	
	SOSG	^1^O_2_	Poor penetration, possibility of photobleaching, high photosensitivity, may react with other ROS, can produce ^1^O_2_	Intracellular, Chloroplasts	
Fluorescent protein biosensors	roGFP1, roGFP2	ROS in general	Reversible, non-invasive, not selective towards specific ROS, enables long-term imaging of ROS, requires plant transformation	Cytosol, Mitochondria, Chloroplasts, ER, Peroxisomes	[219,238]
	rxYFP	ROS in general			
	roGFP-Orp1	H_2_O_2_	Reversible, non-invasive, enables long-term imaging of ROS, requires plant transformation, insensitive to changes in pH	Cytosol, Mitochondria, Chloroplasts	
	cpYFP	O_2_^•−^	Reversible, non-invasive, enables long-term imaging of ROS, requires plant transformation, sensitive to variations in pH	Can be targeted to specific intracellular compartments	
	HyPer	H_2_O_2_	Reversible, non-invasive, enables long-term imaging of ROS, requires plant transformation, sensitive to variations in pH	Cytosol, Chloroplasts, Peroxisomes, Nucleus	

**Table 4:** The ABA-orchestrated strategies developed in shoots and roots during drought.

	Shoot Response (Conservation Strategy)	Root Response (Gain Strategy)
Goal	Arrest growth to limit transpiration surface area and preserve energy	Maintain apex elongation to access deep moist soil (hydrotropism)
ABA action	High accumulation Closing the stomata, inhibition of PM H^+^-ATPase	Spatio-temporal regulation of accumulation Activation of PM H^+^-ATPase in the apex by low concentrations
Cell wall elasticity	Stiffening Increase in CW yield threshold (Y)	Loosening Increase in CW extensibility (*ϕ*)
Apoplast pH	Alkalization pH ~6.0	Acidification in the apex zone pH ~4.5–5.0

## Data Availability

No new data were created or analyzed in this study.
